# Sox7, Sox17, and Sox18 Cooperatively Regulate Vascular Development in the Mouse Retina

**DOI:** 10.1371/journal.pone.0143650

**Published:** 2015-12-02

**Authors:** Yulian Zhou, John Williams, Philip M. Smallwood, Jeremy Nathans

**Affiliations:** 1 Departments of Molecular Biology and Genetics, Johns Hopkins University School of Medicine, Baltimore, Maryland, 21205, United States of America; 2 Department of Neuroscience, Johns Hopkins University School of Medicine, Baltimore, Maryland, 21205, United States of America; 3 Department of Ophthalmology, Johns Hopkins University School of Medicine, Baltimore, Maryland, 21205, United States of America; 4 Howard Hughes Medical Institute, Johns Hopkins University School of Medicine, Baltimore, Maryland, 21205, United States of America; Children's Hospital Boston, UNITED STATES

## Abstract

Vascular development and maintenance are controlled by a complex transcriptional program, which integrates both extracellular and intracellular signals in endothelial cells. Here we study the roles of three closely related *SoxF* family transcription factors–*Sox7*, *Sox17*, and *Sox18* –in the developing and mature mouse vasculature using targeted gene deletion on a mixed C57/129/CD1 genetic background. In the retinal vasculature, each *SoxF* gene exhibits a distinctive pattern of expression in different classes of blood vessels. On a mixed genetic background, vascular endothelial-specific deletion of individual *SoxF* genes has little or no effect on vascular architecture or differentiation, a result that can be explained by overlapping function and by reciprocal regulation of gene expression between *Sox7* and *Sox17*. By contrast, combined deletion of *Sox7*, *Sox17*, and *Sox18* at the onset of retinal angiogenesis leads to a dense capillary plexus with a nearly complete loss of radial arteries and veins, whereas the presence of a single *Sox17* allele largely restores arterial identity, as determined by vascular smooth muscle cell coverage. In the developing retina, expression of all three *SoxF* genes is reduced in the absence of Norrin/Frizzled4-mediated canonical Wnt signaling, but *SoxF* gene expression is unaffected by reduced VEGF signaling in response to deletion of *Neuropilin1* (*Npn1*). In adulthood, *Sox7*, *Sox17*, and *Sox18* act in a largely redundant manner to maintain blood vessel function, as adult onset vascular endothelial-specific deletion of all three *SoxF* genes leads to massive edema despite nearly normal vascular architecture. These data reveal critical and partially redundant roles for *Sox7*, *Sox17* and *Sox18* in vascular growth, differentiation, and maintenance.

## Introduction

Blood vessels are tubular structures composed of vascular endothelial cells (ECs) and mural cells (MCs). They can be divided into arteries, veins, and capillaries according to their anatomy, architecture, and blood oxygen content. Various molecular and cellular specializations, especially those affecting vascular permeability, distinguish blood vessels from different tissues [1,2]. During development, the predominant mechanism of vascular growth is the production of new blood vessels from existing vessels (angiogenesis). This process is orchestrated by endothelial tip cells, which are located at the vascular front and are followed by proliferating stalk cells [3]. Tip cells sense pro- and anti-angiogenic signals, form polarized filopodia, and migrate toward hypoxic tissue [4].

Angiogenesis requires the integration of multiple extracellular signals. In CNS angiogenesis, the specification of tip and stalk cells is balanced by two major signaling pathways: high levels of VEGF signaling induce Dll4 production by tip cells, which activates Notch signaling in the adjacent ECs to confer stalk cell identity [5]. In addition, canonical Wnt signaling is required in developing ECs for vascular invasion into the CNS. In the absence of Wnt or Norrin ligands expressed by the target tissue or in the absence of the Frizzled (Fz) receptor, Lrp5/6 co-receptors, Gpr124/Tspan12 co-activators, or beta-catenin effector expressed by ECs, CNS vascular invasion and vascular network formation are aborted, despite high levels of VEGF produced by the hypoxic target tissue [6,7]. In earlier work, we found that expression of the HMG-box transcription factor Sox17 in retinal veins, arterioles, and capillaries and in immortalized retinal EC lines is dependent on Norrin/Fz4 signaling [8,9]. We also found that constitutive production of Sox17 following viral transduction of the Sox17 coding sequence could rescue the cell migration and filopodial extension defects of *Fz4* ^*-/-*^ retinal ECs cultured on Matrigel [8]. These experiments implicated Sox17 as part of a larger transcriptional program of EC differentiation controlled by canonical Wnt signaling.

Sox17, along with Sox7 and Sox18, belongs to the SoxF branch of the Sox (SRY related-HMG box) family of transcription factors [10]. SoxF family members have been implicated in regulating blood and lymphatic vascular development and arterial-venous identity in a partially redundant fashion [11–13]. In mice, *Sox17* and *Sox18* act together in embryonic and postnatal angiogenesis [14–15], and in zebrafish, double knockdown of *Sox7* and *Sox18* alters arterio-venous specification [16–18]. In mice, dominant *Sox18* mutations, such as the naturally occurring *ragged* (*Ra*) alleles, causes defects in lymphatic vascular development [19,20]. In humans, truncating mutations that eliminate the trans-activation domain or point mutations that alter in the DNA-binding domain of Sox18 cause, respectively, dominant and recessive hypotrichosis-lymphoedema-telangectasia syndrome [21].

One of the most intriguing aspects of SoxF function in mice is the extensive redundancy between family members and the related observation of large genetic-background differences in the severity of *SoxF* mutations. These phenomena emerged with the discovery that targeted deletion of *Sox18* produced only a mild hair phenotype when examined on a mixed genetic background [22]. The contrast between this mild phenotype and the severe embryonic edema caused by dominant *Sox18* mutations of the *Ra* allelic series led Downes and Koopman [23] to predict that: (a) the *Ra* alleles were acting in a dominant negative fashion to broadly inhibit the function of multiple SoxF proteins and (b) in the absence of *Sox18*, *Sox7* and/or *Sox17* could provide compensatory function. Subsequent experiments have confirmed both predictions. Strikingly, when *Sox18* ^*-/-*^ mutants were examined on a pure C57BL/6 background, the severity of the phenotype was greatly enhanced compared to a mixed genetic background, and there was a concomitant loss of *Sox7* and/or *Sox17* expression in embryonic EC territories where *Sox18* is normally expressed [22,24]. Analogous experiments with *Sox17* ^*-/-*^ mice, showed more severe embryonic vascular defects on a pure C57BL/6 background compared to a mixed genetic background and permitted a detailed dissection of *Sox17* function during retinal vascular development using EC-specific deletion of a condition *Sox17* knockout allele [25]. The experiments of Corada et al. [25] demonstrated that Sox17 is a critical regulator of arterial identity and they placed Sox17 upstream of Notch signaling and downstream of canonical Wnt signaling.

Here we report a comprehensive genetic analysis of all three *SoxF* family members on a mixed genetic background using the mouse retina as the main angiogenesis model [26]. Using conventional and/or conditional alleles for *Sox7*, *Sox17*, and *Sox18*, together with immunolocalization of each SoxF protein, we define the retinal vascular defects associated with EC-specific deletion of each *SoxF* gene, all pairwise combinations of *SoxF* genes, and all three *SoxF* genes. We also identify compensatory patterns of *SoxF* gene expression in ECs that likely contribute to the extensive functional redundancy within the *SoxF* family.

## Results

### *Sox7*, *Sox17*, and *Sox18* are expressed in endothelial cells in distinctive patterns

**Fig 1:**
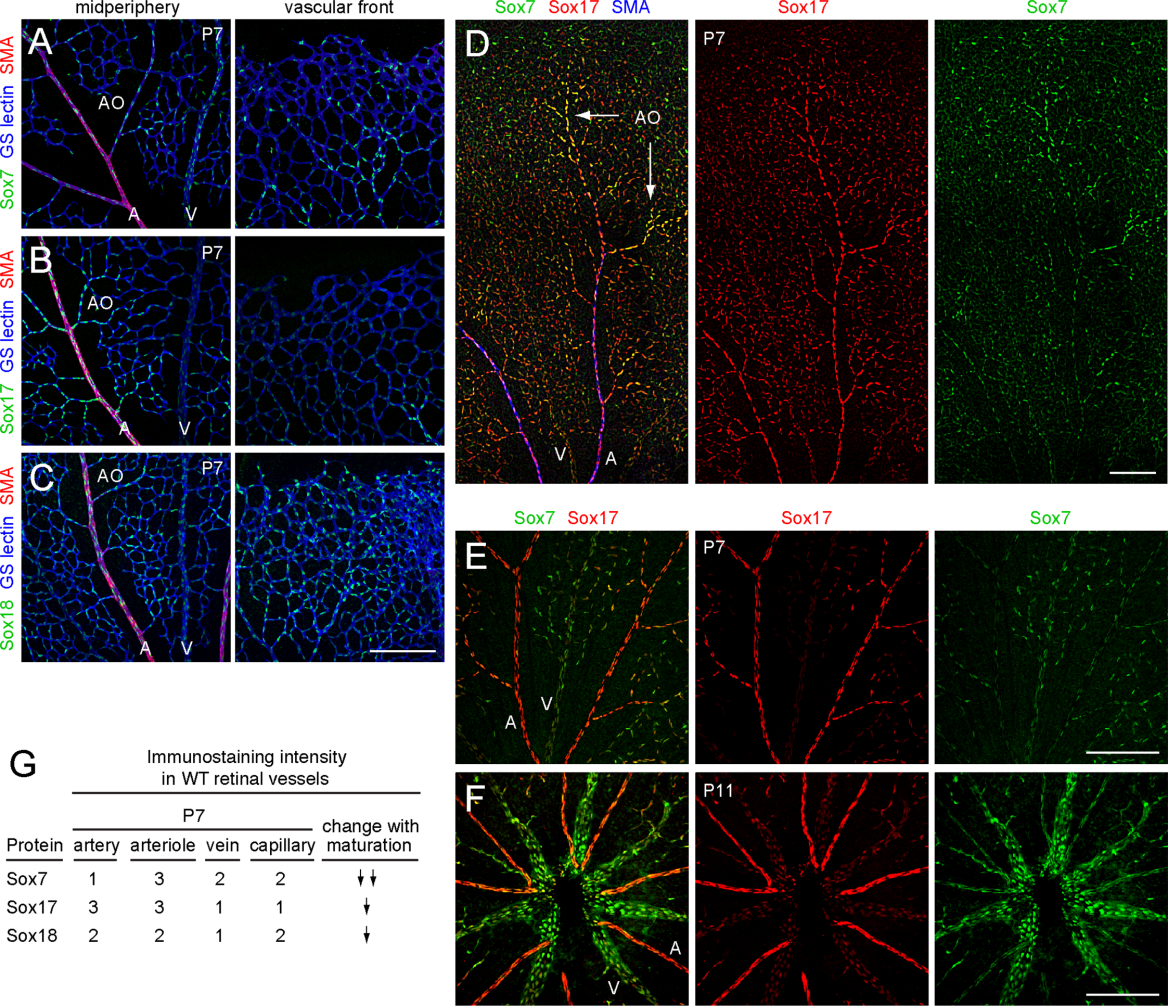
Differential expression of Sox7, Sox17, and Sox18 in the retinal vasculature. (A-C) *Sox7* (A), *Sox17* (B), and *Sox18* (C) are expressed in ECs as determined by anti-Sox7, anti-Sox17 and anti-Sox18 immunostaining of P7 retinas. All retina images in this study are flat mounts. GS-lectin staining marks all blood vessels and anti-smooth muscle actin (SMA) marks arteries. The left panels were imaged in the mid-peripheral retina and the right panels were imaged near the front of the growing vascular plexus; the front is at the top of each image. Scale bar, 200 μm. (D) Mid-periphery of a P7 retina stained with anti-Sox7 (green) and anti-Sox17 (red). Sox17 is abundant in arteries and arterioles. Sox7 is abundant in arterioles but not arteries. A, artery; AO, arteriole; V, vein. Scale bar, 200 μm. (E,F) Mid-periphery of a P7 retina (E) and center of a P11 retina (F) stained with anti-Sox7 (green) and anti-Sox17 (red). Sox17 is abundant in radial arteries but not veins. Sox7 is abundant in veins but not arteries. A, artery; AO, arteriole; V, vein. Scale bar, 200 μm. (G) Summary of immunostaining intensities for SoxF proteins at P7, and extent of reduction in staining intensity in adult retinas (modest reduction, one downward arrow; large reduction, two downward arrows). 1, weak staining; 2, moderate staining; 3, strong staining. Ratings were determined by visual inspection of >100 retinas.

To systematically analyze the function of SoxF family members in retinal angiogenesis, we began by immunolocalizing each protein. In the postnatal day (P)7 mouse retina, each SoxF protein accumulates specifically in EC nuclei with a distinctive pattern of accumulation in arteries, arterioles, veins, and capillaries (Fig 1A–1C; summarized in Fig 1G). Sox7 accumulates to relatively high levels in arterioles, which we operationally define as the alpha smooth muscle actin (SMA)-negative branches that emerge from larger SMA-positive radial arteries. [SMA marks the vascular smooth muscle cells (vSMCs) that surround radial arteries.] Sox7 accumulates to low levels in arteries, and to intermediate levels in veins and capillaries. Sox17 accumulates to relatively high levels in both arteries and arterioles, and to lower levels in veins and capillaries. Sox18 accumulates to relatively uniform levels throughout the retinal vasculature. The different patterns of Sox7 and Sox17 accumulation are clearly seen by double immunostaining (Fig 1D–1F). In the mid-peripheral retina, we observe a relatively high abundance of Sox7 in arterioles and a relatively high abundance of Sox17 in radial arteries (Fig 1D and 1E). In the central retina, we observe a higher abundance of Sox7 in radial veins compared to radial arteries and the reciprocal pattern for Sox17 (Fig 1F).

The different relative abundances of SoxF family members across different vessel types suggested that these proteins might play distinct roles in retinal angiogenesis. To investigate the role of each SoxF family member in the postnatal retina, we constructed conditional knockout (*CKO*) alleles for *Sox7* and *Sox17*, the two *SoxF* genes that are essential for embryonic development (S1A and S2A Figs) [27]. The *Sox7* ^*CKO*^ and *Sox17* ^*CKO*^ alleles were designed to facilitate cell-autonomous assessment of Cre-mediated recombination: the *Sox7* and *Sox17* coding exons and adjacent transcription termination sequences were flanked by *loxP* sites, and coding sequences for a reporter [triple heamaglutinin (3xHA) tagged cyan fluorescent protein (CFP) for *Sox7* ^*CKO*^ and human placental alkaline phosphatase (AP; a GPI-linked plasma membrane protein) for *Sox17* ^*CKO*^] were inserted distal to the 3’ *loxP* site. Thus, Cre-mediated deletion of *Sox7* coding sequences leads to expression of CFP-3xHA, and Cre-mediated deletion of *Sox17* coding sequences leads to expression of AP (S1B Fig and S2B and S2C Fig).

Embryos in which *Sox7* was constitutively deleted (*Sox7* ^*-/-*^) or deleted specifically in endothelial and hematopoietic cells (*Sox7* ^*CKO/CKO*^ *;Tie2-Cre*) died by embryonic day (E)11.5 (S3A Fig). Although gross vascular development in *Sox7* ^*CKO/CKO*^ *;Tie2-Cre* embryos was largely unperturbed at E10.5 (S3B Fig), upon closer inspection, we observed a reduction in the width of the left dorsal aorta and a reduction in its coverage by vSMCs (S3C Fig). This defect occurs despite expression of *Sox17* in ECs in the dorsal aorta (S3C Fig), suggesting that *Sox17* cannot compensate for the loss of *Sox7* in this context.

**Fig 2:**
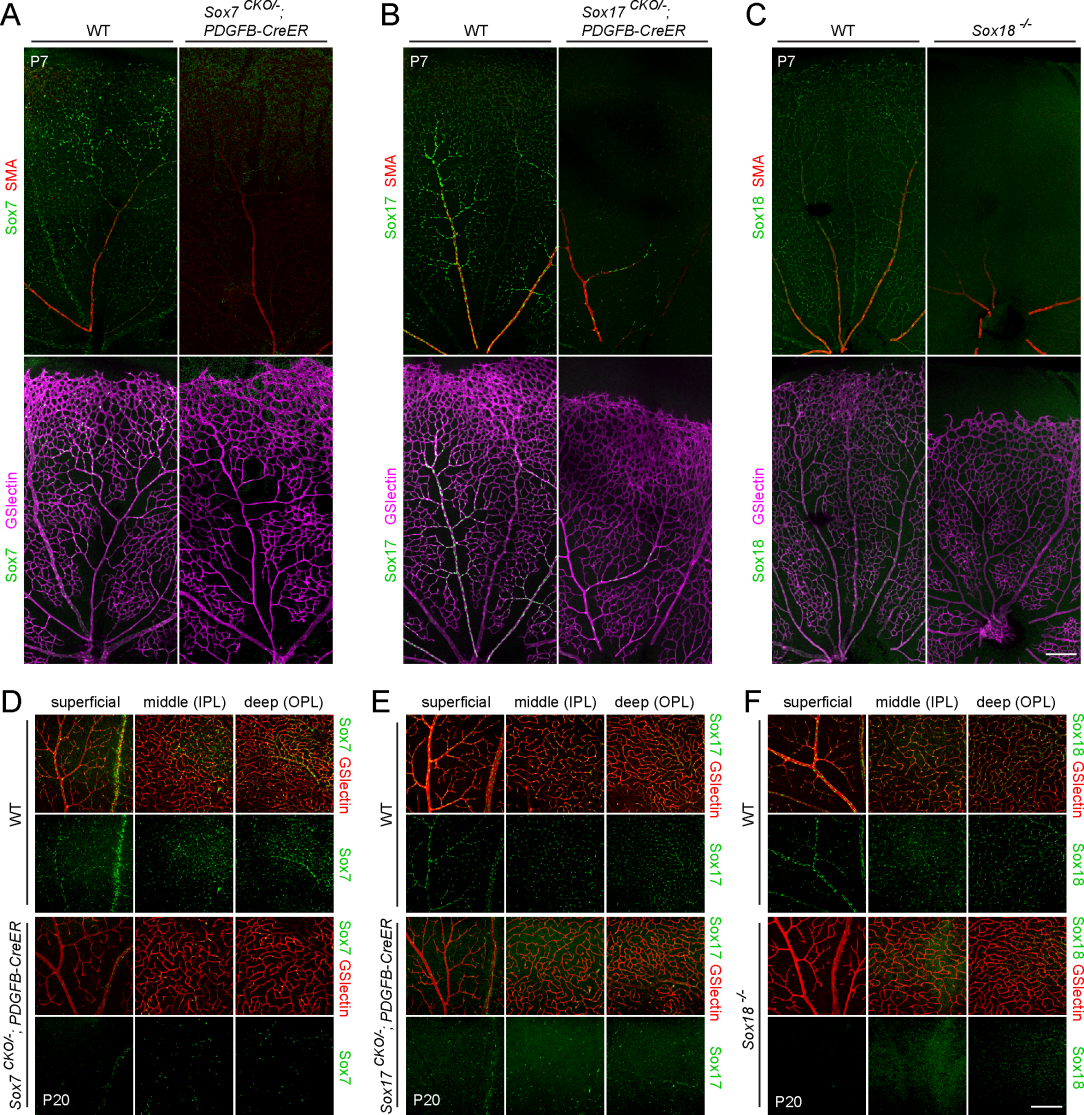
Deletion of Sox7, Sox17, or Sox18 has little or no affect on vascular development in the retina. (A-C) P7 retinal vasculature in WT (A-C, left panels), *Sox7* ^*CKO/-*^ *;Pdgfb-CreER* (A, right panels), *Sox17* ^*CKO/-*^ *;Pdgfb-CreER* (B, right panels), and *Sox18* ^*-/-*^ (C, right panels) shown by GS-lectin staining, with vSMC coverage of radial arteries visualized with anti-SMA. 50 μg 4HT was given at P2. Scale bar, 200 μm. (D-F) P20 retinal vasculature in WT (D-F, upper panels), *Sox7* ^*CKO/-*^ *;Pdgfb-CreER* (D, bottom panels), *Sox17* ^*CKO/-*^ *;Pdgfb-CreER* (E, bottom panels), and *Sox18* ^*-/-*^ (F, bottom panels) shown by GS-lectin staining. All three vascular layers are present in the knockout retinas. Anti-Sox7, anti-Sox17, and anti-Sox18 immunostaining show antibody specificity (the immunostaining signal is present in WT and absent in knockout retinas stained in parallel), and conditional knockout efficiency by CreER. 50–100 μg 4HT was given at P2-4. IPL, inner plexiform layer. OPL, outer plexiform layer. Scale bar, 200 μm.

To circumvent the problem of embryonic lethality, we generated *Sox7* ^*CKO/-;*^ *PDGFB-CreER* and *Sox17* ^*CKO/-*^ *;PDGFB-CreER* mice to delete *Sox7* and *Sox17*, respectively, in vascular ECs by administering 4-hydroxytamoxifen (4HT) starting at approximately postnatal day (P)2. Under these conditions, Cre-mediated recombination of the *CKO* allele occurs in the vast majority of retinal ECs as determined by the appearance of the CFP-3xHA and AP reporters and by the loss of Sox7 and Sox17 immunoreactivity (Fig 2, and S2B–S2D Fig). Following the loss of *Sox7* or *Sox17* at ~P2, we observed no defects in the growth, density, or lamination of the various vessel types or in the recruitment of vSMCs to radial arteries in retinas at either P7 or P20 (Fig 2A, 2B, 2D and 2E). Similarly, *Sox18* ^*-/-*^ mice, which are healthy and fertile, showed normal retinal vascular structure (Fig 2C and 2F).

The anti-Sox7, anti-Sox17, and anti-Sox18 antibodies appear to be specific for their target proteins because the immunostaining signal was abolished when the corresponding gene was knocked out (Fig 2). In these experiments, the WT and knockout retinas were processed, stained, and imaged in parallel with matched reagents and protocols. Thus, for each SoxF factor, the paired immunostaining images of WT and KO retinas are directly comparable. These experiments validate the specificity of the anti-Sox7, anti-Sox17, and anti-Sox18 antibodies, and they show that loss of individual SoxF factors has virtually no effect on retinal vascular development on the C57/129/CD1 mixed genetic backgrounds used here.

### *Sox7*, *Sox17*, and *Sox18* play redundant roles during vascular development

**Fig 3:**
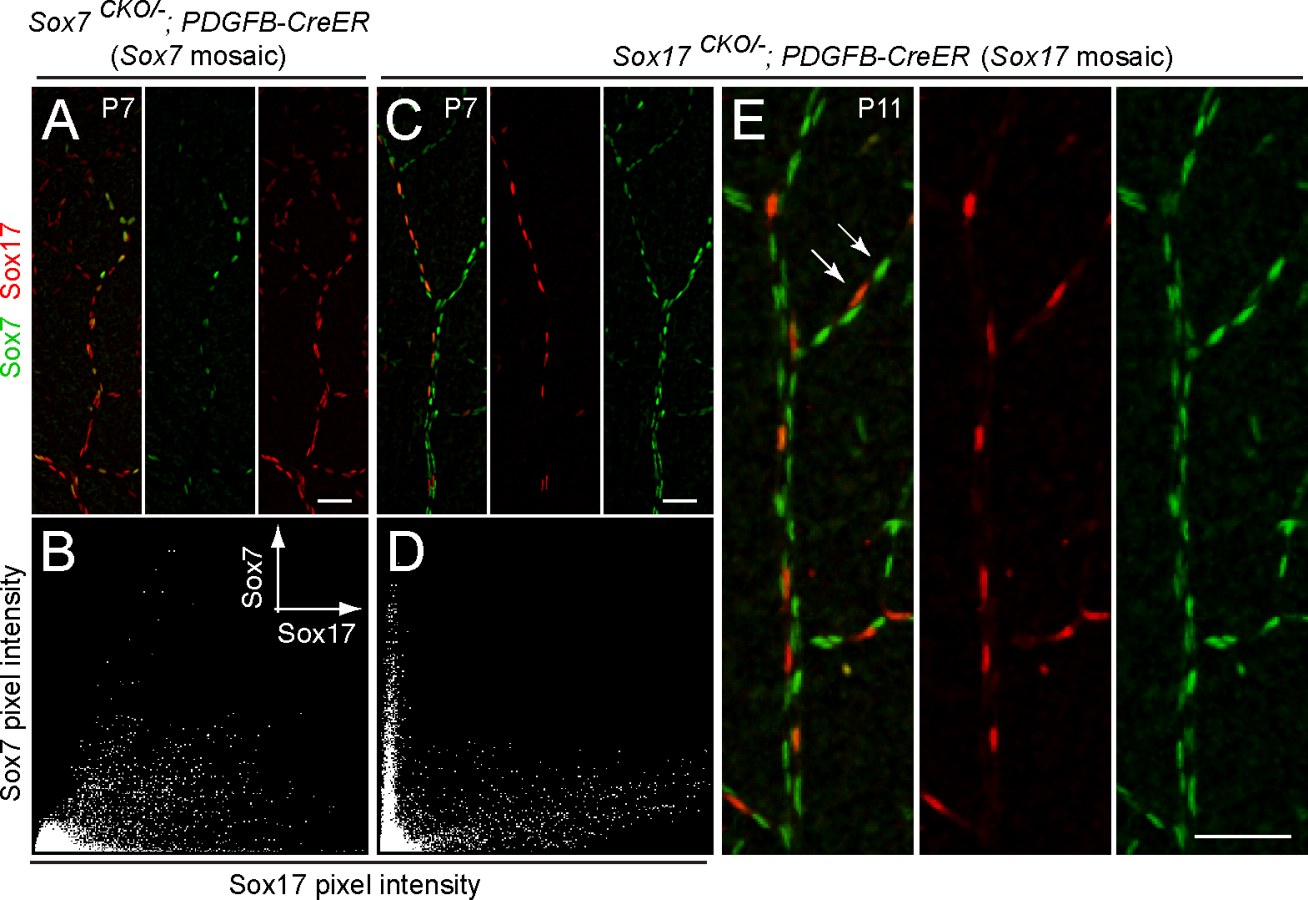
Sox7 expression is up-regulated in Sox17^-/-^ ECs in mosaic Sox17^CKO/-^;Pdgfb-CreER retinal vasculature. (A-E) Flat mount retinas stained with anti-Sox7 and anti-Sox17 from *Sox7* ^*CKO/-*^ *;Pdgfb-CreER* mice at P7 (A,B), and from *Sox17* ^*CKO/-*^ *;Pdgfb-CreER* mice at P7 (C,D) and at P11 (E), following 50 μg 4HT at P2. (B,D) Scatterplots of Sox7 pixel intensity vs. Sox17 pixel intensity; (B) and (D) correspond to (A) and (C), respectively. EC nuclei in *Sox17* ^*CKO/-*^ *;Pdgfb-CreER* retinal vasculature express either Sox17 (i.e. unrecombined Sox17+ cells; lower white arrow in E) or Sox7 (recombined Sox17- cells; upper white arrow in E). Scale bar, 50 μm.

The failure to observe a retinal vascular phenotype in the absence of individual *SoxF* family members suggested that there might be functional compensation by one or both of the remaining family members. The following observations support this model. First, simultaneous inactivation of *Sox17* and *Sox18* in early postnatal life (in 4HT treated *Sox17* ^*CKO/CKO*^ *; Sox18* ^*-/-*^ *;PDGFB-CreER* mice) led to reduced vascular coverage of the retinal surface, hyper-dense capillaries, enlargement of veins, and a decrease in vSMC coverage of arteries (S4A, S4B and S4D Fig). In contrast, when *Sox17* and *Sox18* were eliminated from ECs in adulthood, no defect was seen in the retinal vasculature (S4C Fig). Second, in retinal arteries of *Sox17* ^*CKO/-*^ *;PDGFB-CreER* mice in which the *Sox17* locus was deleted in only a fraction of ECs in early postnatal life, we observed a marked increase in Sox7 levels in Sox17-null ECs as compared with adjacent Sox17-expressing ECs (Fig 3C–3E). This effect persisted into adulthood (data not shown). Performing this analysis with genetically mosaic retinas provides optimal conditions for a comparison between Sox17-expressing and Sox17-null ECs, since the cells are adjacent to one another in the same vessel. Quantification of anti-Sox7 vs. anti-Sox17 immunostaining intensities shows two distinct classes of immunostained nuclei (Fig 3D). In contrast, there was little change in Sox17 levels in Sox7-null retinal ECs from *Sox7* ^*CKO/-*^ *;PDGFB-CreER* mice in which the *Sox7* locus was similarly deleted in a fraction of ECs (Fig 3A and 3B).

**Fig 4:**
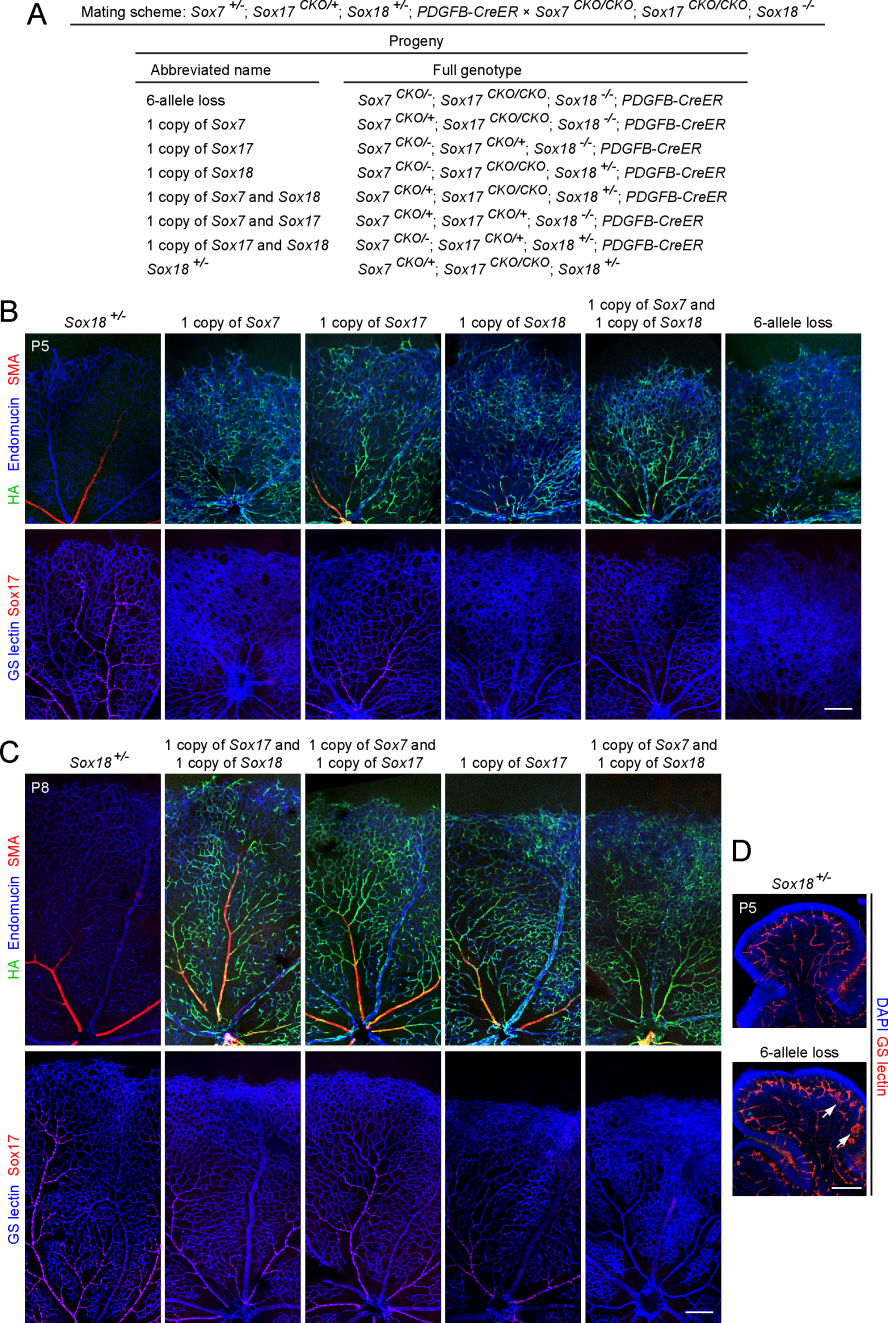
Combined loss of Sox7, Sox17, and Sox18 in ECs alters retinal vasculature. (A) Summary of the triple *SoxF* cross and the resulting progeny with abbreviations for their genotypes. (B, C) Analysis of retinal vasculature in *SoxF* mutants at P5 (B) and P8 (C). Top panels, retina flat mounts immunostained with anti-endomucin, anti-SMA and anti-HA. Bottom panels, retina flat mounts stained with GS-lectin and anti-Sox17. Anti-HA and anti-Sox17 staining assesses CreER-mediated recombination efficiency. The retinal vasculature in *Sox18* ^*+/-*^ is indistinguishable from WT. The mutants that lack *Sox17* and either one or both copies of *Sox7* and *Sox18* show greatly reduced vSMC coverage of radial arteries with increased capillary density. 50–100 μg 4HT was given at P0 and P2. Scale bar, 200 μm. (D) Cerebellum sections from *Sox7* ^*CKO/-*^ *;Sox17* ^*CKO/CKO*^ *;Sox18* ^*-/-*^ *;Pdgfb-CreER* and *Sox18* ^*+/-*^ control at P5, following 50–100 μg 4HT at P0 and P2. Vascular disorganization (arrows) is seen in *Sox7* ^*CKO/-*^ *;Sox17* ^*CKO/CKO*^ *;Sox18* ^*-/-*^ *;Pdgfb-CreER*. Scale bar, 200 μm.

To extend this analysis to the full range of *Sox7*, *Sox17*, and *Sox18* allelic combinations, we set up a cross between *Sox7* ^*+/-*^; *Sox17* ^*CKO/+*^; *Sox18* ^*+/-*^ *; PDGFB-CreER* and *Sox7* ^*CKO/CKO*^; *Sox17* ^*CKO/CKO*^; *Sox18* ^*-/-*^ mice (Fig 4A). This cross was designed so that the genotypes of the progeny would be equally likely to possess the following four pairs of alternate alleles: (1) *Sox7* ^*CKO/+*^ or *Sox7* ^*CKO/-*^, (2) *Sox17* ^*CKO/+*^ or *Sox17* ^*CKO/CKO*^, (3) *Sox18* ^*+/-*^ or *Sox18* ^*-/-*^, and (4) the *PDGFB-CreER* transgene or no *CreER*. Thus, the 16 progeny genotypes included those that conferred 4HT-induced loss of the following WT alleles: (1) all 6 *SoxF* alleles, (2) 5/6 *SoxF* alleles with retention of a single allele of *Sox7*, *Sox17*, or *Sox18*, (3) 4/6 *SoxF* alleles with retention of any of the three possible pairs of single alleles (*Sox7* and *Sox17*, *Sox7* and *Sox18*, or *Sox17* and *Sox18*), or (4) only a single *Sox18* allele with retention of the remaining five *SoxF* alleles, thereby providing a phenotypically normal littermate control. Abbreviated names for these various progeny genotypes are listed in Fig 4A.

The constraints of embryonic lethality associated with homozygosity for *Sox7* and *Sox17* mutations, together with our observation that *Sox7* ^*+/-*^ *;Sox17* ^*+/-*^ mice were produced far below the expected Mendelian frequency (S3A Fig), limited the number and type of *SoxF* null alleles that could be combined in the parents. As a consequence of these constraints, eliminating *Sox17* in the F1 progeny required Cre-mediated deletion of two alleles (starting from *Sox17* ^*CKO/CKO*^) instead of one (starting from *Sox17* ^*CKO/-*^), and several genotypes required three Cre-mediated somatic deletion events to attain the final EC-specific genotype (Fig 4A). Conveniently, the efficiency of recombination of the *Sox17* ^*CKO*^ allele was found to be extremely high (e.g. Fig 2B). However, the efficiency of Cre-mediated recombination of the single *Sox7* ^*CKO*^ allele (in a *Sox7* ^*CKO/-*^ or *Sox7* ^*CKO/+*^ context) was substantially lower, perhaps reflecting the larger distance between *loxP* sites in the *Sox7* ^*CKO*^ allele compared to the *Sox17* ^*CKO*^ allele. Therefore, the *Sox7* ^*CKO*^ to *Sox7* ^*CFP-3xHA*^ conversion was monitored in most experiments by immunostaining for the CFP-3xHA reporter. In order to detect the *Sox7* ^*CKO*^ to *Sox7* ^*CFP-3xHA*^ conversion in the presence of a constitutive *Sox7* null allele, we generated a constitutive *Sox7* null allele that lacked the CFP-3xHA reporter (*Sox7* ^*-*^; S2A Fig). Thus, the anti-HA staining in Fig 4 monitors the loss of *Sox7* as determined by the *Sox7* ^*CKO/-*^ to *Sox7* ^*CFP-3xHA/-*^ conversion.

In vasculature with only one allele of *Sox7* and/or *Sox18*, there is substantial EC hyperplasia and vSMC coverage of arteries is largely absent at both P5 and P8 (Fig 4B and 4C). The presence of one allele of *Sox17* with or without one copy of *Sox7* and/or *Sox18* largely suppressed the EC hyperplasia and supported vSMC coverage. The nearly absent arterial vSMC coverage in vasculature lacking *Sox17* and retaining only one allele of *Sox7* and/or *Sox18* is in contrast with the nearly normal vSMC coverage observed in vasculature retaining only a single allele of *Sox17*, implying a more prominent role for *Sox17* in arterial differentiation compared to *Sox7* and/or *Sox18*. Interestingly, vSMC coverage is essentially normal in the complete absence of *Sox17* if there is a full complement of *Sox7* and *Sox18* alleles (Fig 2), implying that, when expressed at sufficient levels, *Sox7* and/or *Sox18* can largely or fully compensate for the absence of *Sox17*. This compensation could reflect up-regulation of *Sox7* (Fig 3).

**Fig 5:**
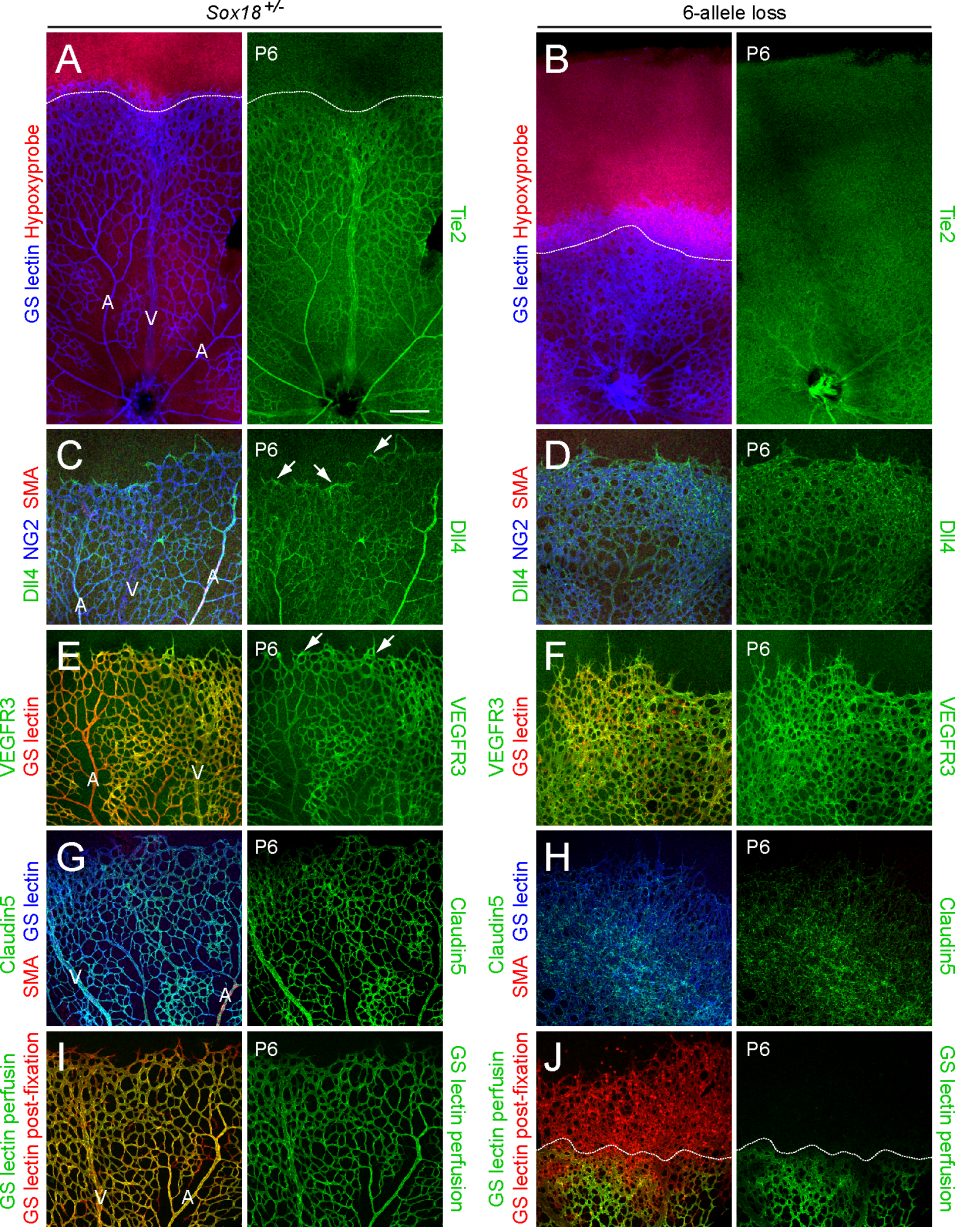
Excess tip cell production in the Sox7, Sox17, and Sox18 EC-specific triple KO (‘6-allele loss’) at P6. (A-J) Retina flat mounts from control *Sox18* ^*+/-*^ (A, C, E, G, I) and 6-allele loss *Sox7* ^*CKO/-*^ *;Sox17* ^*CKO/CKO*^ *;Sox18* ^*-/-*^ *;Pdgfb-CreER* (B, D, F, H, J) mice at P6. In (I) and (J), retinas were labeled with Alexa488 GS-lectin (green) via intracardiac injection and Alexa595 GS-lectin (red) after postfixation and permeabilization. The dashed lines delineate the boundary between hypoxia/normoxia in (A and B), and perfused/non-perfused vessels in (J). In control retinas, most tip cells (arrows) express higher levels of Dll4 (C) and VEGFR3 (E) than adjacent capillaries. The larger zone of hypoxia in (B) compared to (A) reflects the retarded growth of the vascular plexus. 50–100 μg 4HT was given at P0 and P2. A, artery; V, vein. Scale bar, 200 μm.

Early postnatal EC-specific deletion of all six *SoxF* alleles leads to the most severe defects in retinal vascular development, with variably retarded radial migration, severe EC hyperplasia in veins and capillaries, and a loss of vSMC coverage of radial arteries (Fig 4B and 4C, Fig 5). In the absence of 4HT treatment, *Sox7* ^*CKO/-*^; *Sox17* ^*CKO/CKO*^; *Sox18* ^*+/-*^ *; PDGFB-CreER* mice were healthy and fertile, with normal retinal vascular development.

Since the severity of the vascular phenotype for each genotype varied somewhat depending on the efficiency of Cre-mediated recombination, Fig 4B and 4C show examples of the most severely affected retinas for each genotypes, i.e. those with nearly complete recombination for *Sox17* (as measured by anti-Sox17 immunostaining) and >50% recombination for *Sox7* (as measured by anti-3×HA staining). For a given genotype and degree of Cre-mediated recombination, phenotypic variability was relatively small, implying that animal-to-animal variation in the random segregation of allelic variants from the mixed genetic background was not a major sources of phenotypic variability. A total of 6–12 retinas were analyzed for each genotype at both time points. In addition to the retinal vascular phenotypes, disorganization and hyperplasia of the developing cerebellar vasculature was observed in mice with EC-specific deletion of all *SoxF* alleles and, in milder form, in mice deficient for both *Sox17* alleles and one *Sox18* allele (Fig 4D, S4E Fig). Taken together, these data imply that *Sox17* plays a major role and *Sox7* and *Sox18* are of lesser importance in retinal vascular development.

### Sox7, Sox17, and Sox18 regulate endothelial cell differentiation

To further characterize the retinal vascular phenotype resulting from the loss of all six *SoxF* alleles, we visualized endothelial stalk vs. tip cell phenotypes by Tie2, Dll4, and VEGFR3 immunostaining in P6 retina flat mounts. In phenotypically normal controls (*Sox18* ^*+/-*^), endothelial tip cells at the front of the vascular plexus expressed elevated levels of the Notch ligand Dll4 and VEGF receptor 3 (VEGFR3) and reduced levels of the Angiopoetin receptor Tie2 (Fig 5A, 5C and 5E). In the 6-allele loss retinal vasculature, it was not possible, based on immunstaining, to distinguish between tip cell-like ECs at the vascular front (operationally defined as ECs with elevated levels of Dll4 and VEGFR3 and reduced levels of Tie2) and stalk-like ECs (operationally defined as ECs with lower levels of Dll4 and VEGFR3 and elevated levels of Tie2). In the 6-allele loss retina, Tie2 levels were reduced throughout the retinal vasculature at P6, and Dll4 and VEGFR3 were present throughout the growing plexus and were not enriched at the vascular front (Fig 5B, 5D and 5F). In the 6-allele loss retina there was little or no change in vascular coverage by NG2^+^ mural cells (Fig 5C and 5D), and the endothelial tight junction marker Claudin5 was present in a pattern that reflected the disorganized vascular architecture (Fig 5G and 5H).

The dense and disorganized vascular plexus in the 6-allele loss retina exhibited reduced perfusion when patent vessels, visualized by intracadiac injection of GS lectin, were compared to the entire vascular plexus, visualized with GS-lectin labeling following tissue fixation and permeabilization (Fig 5J). This is in contrast to the phenotypically WT control, which shows uniform perfusion of the entire plexus, except for the single row of tip cells at the vascular front (Fig 5I). Consistent with the reduced perfusion of the 6-allele loss vascular plexus, histochemical localization of tissue hypoxia based on the accumulation of pimonidazole (Hypoxyprobe) [28], revealed substantial overlap between the region of hypoxic peripheral retina and the growing vascular plexus, whereas in the control retina these territories exhibit a smaller overlap (Fig 5A and 5B). It is likely that in the 6-allele loss retina compromised vascular perfusion leads to an elevation in retina-derived VEGF, which promotes the increase in vascular density.

### Loss of Sox7, Sox17, and Sox18 in adult vasculature causes subcutaneous edema

**Fig 6:**
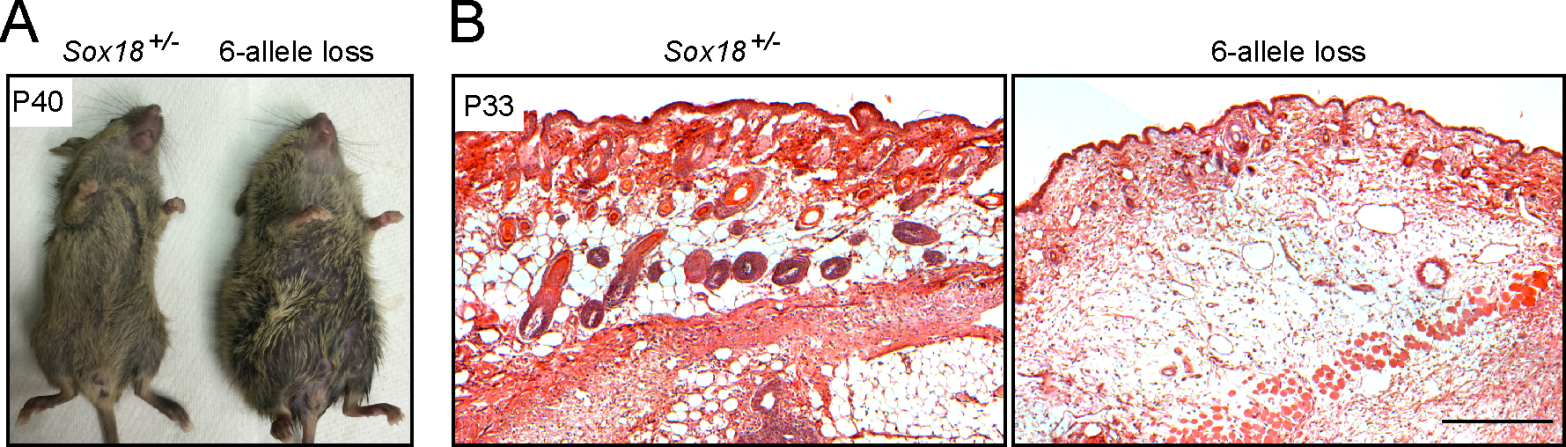
Sox7, Sox17, and Sox18 EC-specific triple KO in adulthood leads to widespread peripheral edema. (A) A representative *Sox7* ^*CKO/-*^ *;Sox17* ^*CKO/CKO*^ *;Sox18* ^*-/-*^ *;Pdgfb-CreER* mouse (right) that had received 2–3 mg tamoxifen at P21, P25 and P29 developed an edematous phenotype at P40. The control *Sox18* ^*+/-*^ littermate (left) that had received the same tamoxifen treatment was unaffected. (B) Haematoxylin and eosin-stained sections of the abdominal wall from a control (left panel) mouse and a *Sox7* ^*CKO/-*^ *;Sox17* ^*CKO/CKO*^ *;Sox18* ^*-/-*^ *;Pdgfb-CreER* mouse (right panel) at P33, following 2–3 mg tamoxifen at P21, P23 and P25. Dermal edema is present in the 6-allele loss skin. Scale bar, 200 μm.

Expression of *Sox7*, *Sox17*, and *Sox18* persists in the adult retinal vasculature (Fig 2E, 2F and S2D Fig), suggesting a continuing role for SoxF factors in mature ECs. Although *Sox7* expression is reduced in the adult retinal vasculature, a potential role for *Sox7* in mature ECs that lack *Sox17* seemed possible since Sox7 levels increase when Sox17 is eliminated in both the developing and adult retinal vasculature (Fig 3). To explore the role of SoxF factors in the adult vasculature, we treated *Sox7* ^*CKO/-*^ *;Sox17* ^*CKO/CKO*^ *;Sox18* ^*-/-*^ *;PDGFB-CreER* mice with high doses of tamoxifen from P21 to P29 to generate an EC-specific 6 allele loss genotype. Strikingly, 13 of 14 of these mice developed massive edema 10 to 30 days after tamoxifen treatment, compared to zero of 14 of their tamoxifen treated *Sox7* ^*CKO/-*^ *;Sox18* ^*-/-*^ littermates (Fig 6). Except for a subtle enlargement of retinal veins, the vascular architecture and vascular integrity of the edematous 6-allele knockout mice appeared to be grossly normal in multiple tissues including the skin (S5A and S5B Fig, and data not shown).

A similar experiment conducted with *Sox7* ^*CKO/+*^ *;Sox17* ^*CKO/CKO*^ *;Sox18* ^*-/-*^ *;PDGFB-CreER* mice (i.e., one allele of *Sox7*) produced edema in four of four mice 20–50 days after tamoxifen treatment, indicating that a single *Sox7* allele in 5-allele loss mice delays but does not ultimately rescue the edema phenotype. In contrast, only one of four 5-allele loss mice with a single copy of *Sox18* developed edema 4 months after adult tamoxifen treatment, and zero of four 5-allele loss mice with a single copy of *Sox17* developed edema up to 5-months after adult tamoxifen treatment (data not shown). Although no change in vascular permeability was detected by the Miles assay, which measures acute Evans Blue dye extravasation into tissues following an intravenous dye injection (S5C Fig), it seems likely that the edema phenotype reflects a subtle defect in vascular permeability that leads to an altered equilibrium between intra- and extravascular fluid levels.

Previous studies showed that *Sox18* ^*-/-*^ embryos on a pure C57 background developed extensive edema with defects in pericyte coverage and multiple hemorrhages, whereas *Sox18* ^*-/-*^ mice on a mixed 129/CD1 background exhibited no discernable phenotype [14,22,29]. Moreover, Francois et al [22] observed *Sox18* expression in embryonic lymphatic vessels but they did not detect *Sox18* expression in the lymphatic endothelium postnatally, implying that Sox18 is not required for maintenance of lymphatic vessels. In agreement with the observations of Matsui et al [14] we find no evidence for edema in *Sox18* ^*-/-*^ mice on the mixed background used here. The edematous phenotype induced by adult deletion of all *SoxF* genes appears to be caused by defects in the blood vasculature rather than the lymphatic vasculature because the *CreER* driver used in these experiments, *Pdgfb-CreER*, is active in blood but not lymphatic vasculature, as determined by testing it with the highly recombinogenic *Hprt* ^*LSL–tdT*^ reporter (S6 Fig) [30]. Taken together, these data indicate that *Sox17* and *Sox18* play an essential and largely redundant role in the maintenance of fluid balance across the adult blood vasculature.

### Canonical Wnt signaling but not VEGF signaling regulates *Sox7*, *Sox17*, and *Sox18* expression

**Fig 7:**
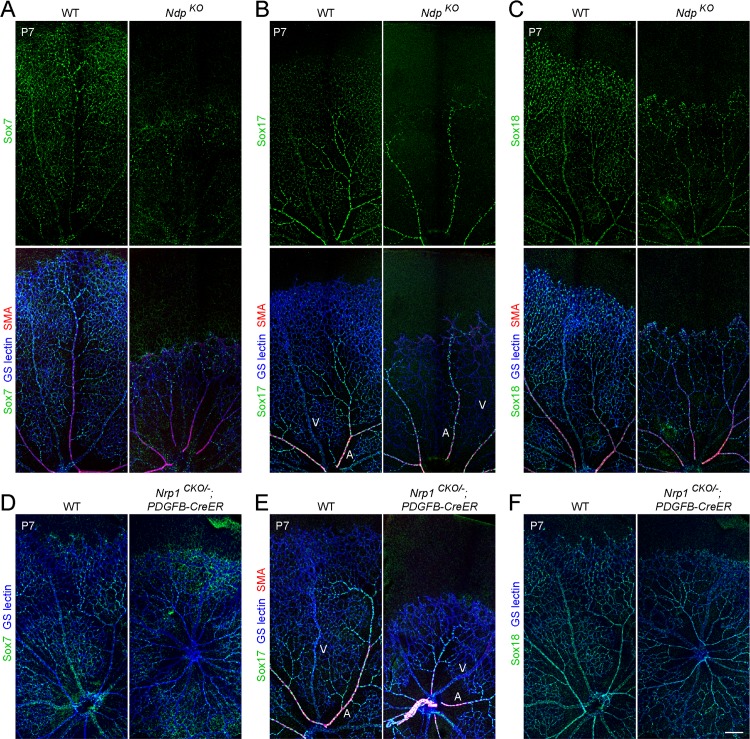
The expression of Sox7, Sox17, and Sox18 is regulated by Norrin signaling but not by VEGF/VEGFR2/NRP1 signaling. (A-F) Anti-Sox7, anti-Sox17, and anti-Sox18 immunostaining in flat-mount retinas from WT (A-F, left panels), *Ndp* ^*KO*^ (A-C, right panels), and *Nrp1* ^*CKO/-*^ *;Pdgfb-CreER* (D-F, right panels) mice at P7. (Panels D and F show the same pair of retinas, double stained with anti-Sox7 and anti-Sox17, with both immunolabeling signals visualized in the green channel for ease of comparison. Some non-specific non-EC immunostaining is seen in the Sox7 immunostained retinas in panel D.) Sox17 levels are dramatically decreased in veins and capillaries in *Ndp* ^*KO*^ (B), while Sox7 and Sox18 levels are modestly decreased in *Ndp* ^*KO*^ (A and C). Loss of *Nrp1* has little or no effect on SoxF protein levels. Arteries and veins are marked for the *Sox17* mutant retinas. Scale bar, 200 μm.

As noted in the introduction, in earlier work, we found that *Sox17* expression in retinal veins, arterioles, and capillaries, and in immortalized retinal EC lines is dependent on canonical Wnt signaling mediated by Norrin/Fz4 [8,9]. Here, we extend this analysis to *Sox7* and *Sox18*, by immunostaining retinas that lack *Ndp*, the gene coding for Norrin. (*Ndp* is X-linked and we refer to both *Ndp* ^*-/-*^ females and *Ndp* ^*-/Y*^ males as *Ndp* ^*KO*^.) In the *Ndp* ^*KO*^ retina at P7, Sox7 and Sox18 levels were moderately decreased, whereas Sox17 was barely detectable in veins, arterioles, and capillaries, while remaining unchanged in radial arteries (Fig 7A–7C). We note that the modest reduction in Sox7 levels in the *Ndp* ^*KO*^ retina may underestimate the magnitude of any direct effect of canonical Wnt signaling on *Sox7* gene expression because, as seen in Fig 3C–3E, there is a compensatory increase in *Sox7* expression upon loss of *Sox17*.

At present, it is unclear which aspects of the angiogenesis and CNS vascular barrier defects associated with loss of Norrin/Fz4 signaling are referable to decreased expression of *SoxF* genes, as we have not observed defects in the blood-retina barrier or the blood-brain barrier in *SoxF* 6-allele loss mice nor have we been able to rescue the *Ndp* ^*KO*^ vascular phenotypes by over-expressing *Sox17* in ECs in vivo (unpublished data). While earlier experiments with retinal vascular ECs in culture indicated that Sox17 acts downstream of Norrin/Fz4 signaling to regulate EC migration [8], other effectors are likely to also be involved in orchestrating the Norrin/Fz4 response in vivo.

Disruption of VEGF/VEGFR2/Neuropilin1 (Nrp1) signaling by deletion of *Nrp1* leads to multiple defects in retinal angiogenesis, including defects in vascular invasion and arborization within the retina [31,32]. We therefore asked whether expression of *SoxF* members is altered in the absence of VEGF/VEGFR2/Neuropilin1 signaling. For these experiments, we studied *Nrp1* ^*CKO/-*^ *;PDGFB-CreER* mice that had received 4HT in early postnatal life, thereby circumventing the early postnatal lethality associated with constitutive loss of *Nrp1*. As shown in Fig 7D–7F, EC-specific deletion of *Nrp1* led to retarded vascular growth but did not alter the expression of *SoxF* family members. Taken together, these observations imply that *SoxF* expression in the retinal vasculature is regulated by Norrin/Fz4 signaling but not by VEGF/VEGFR2/Neuropilin1 signaling.

## Discussion

The widespread existence of gene families has long been an object of interest to biologists, but the functional dissection of homologous genes—both individually and in combination—poses significant technical challenges. In this study, we define overlapping roles for the mouse *SoxF* genes—*Sox7*, *Sox17* and *Sox18*—in vascular growth, differentiation, remodeling, and maintenance, as assessed on a mixed C57/129/CD1 background. The principal results of this study are: (1) each SoxF factor exhibits a distinctive pattern of accumulation among the different classes of retinal blood vessels; (2) different severities of retinal vascular defects are observed among the seven possible combinations of single, double, and triple *SoxF* gene deletions; (3) *Sox17* plays a major role in promoting vSMC coverage of radial arteries; (4) *SoxF* family members play partially redundant roles in retinal vascular remodeling; (5) in retinal ECs, there is a cell-autonomous increase in expression of *Sox7* when *Sox17* is eliminated; (6) *SoxF* family members are required for normal EC function in the adult peripheral vasculature; and (7) in the *Ndp* ^*KO*^ retina, EC expression of all three *SoxF* genes is reduced, implicating canonical Wnt signaling as a broad-based *SoxF* regulator.

### Mechanisms of redundancy in the SoxF gene family

In studying the role of *Sox18* in lymphangiogenesis, Pennisi et al [19], Francois et al [22], and Hosking et al [24] observed severe edema in *Sox18* ^*-/-*^ mutants on a pure C57BL/6 background but only a very mild phenotype on a mixed 129/CD1 background. The mild phenotype on the mixed background was associated with induction of *Sox7* and *Sox17* gene expression at the site of future lymphatic vessel development, and accumulation of Prox1, a lymphatic marker and Sox18 target. The implication from these observations is that lymphatic development is nearly normal on the mixed genetic background because of a compensatory induction of *Sox7* and *Sox17* in lymphatic ECs, where they are not normally expressed. Our experiments with Sox7-specific and Sox17-specific immunostaining in *Sox7* and *Sox17* mosaic vasculatures extend the concept of reciprocal regulation of *SoxF* family members to the developing blood vascular system. Taken together with the observations of Corada et al [25] showing that *Sox17* ^*-/-*^ mice have a severe arterial phenotype on a pure C57BL/6 background, these observations define a genetic mechanism that functions as a buffer to minimize the effects of variation in the expression levels of individual *SoxF* genes. It seems possible that reciprocal regulation among *SoxF* family members could increase the robustness of blood and lymphatic vascular development to both genetic and environmental perturbations.

It would be interesting to explore the molecular basis of the genetic background-specific effects on *SoxF* gene regulation. For example, it might be possible to use congenic mouse lines to identify the genomic regions associated with this regulation and to determine whether genetic variation in or near the *SoxF* genes are responsible or whether the effect segregates with other genomic loci. It would also be interesting to systematically explore the extent to which compensatory changes in gene expression account for other instances of partial or complete functional redundancy among highly homologous gene family members. This phenomenon has been observed in a variety of systems, and it could play a major role in modifying the severity of human disease. For example, individuals with beta-thalassemia due to reduced production of adult beta-globin experience a compensatory induction of the closely related fetal gamma-globin [33]. Higher levels of gamma-globin produce a milder thalassemia phenotype.

### Links between SoxF function and Notch signaling

Previous studies have established a central role for Notch signaling in tip cell to stalk cell conversion [34], and several studies have examined interactions between *SoxF* genes and Notch signaling [35,36]. In ECs in mouse and zebrafish embryos, SoxF proteins act in synergy with RbpJ, the major transcriptional effector of Notch signaling, to activate *Dll4* and promote arterial identity [37]. Additionally, Corada et al [25] showed that Sox17 activates Notch signaling by binding to the promoters of multiple genes in the Notch pathway, and Hermkens et al. [38] showed that over-expression of the Notch intracellular domain rescues the arterial specification defects in *Sox7* null zebrafish, implying that SoxF proteins function upstream of Notch signaling. Interestingly, Notch signaling also suppresses *Sox17* expression, suggesting a feedback loop with Notch as an upstream regulator of *Sox17* [39].

The retinal vascular phenotype reported here in mice with 6-allele *SoxF* loss closely resembles the defects caused by loss of Notch signaling, consistent with the established connection between SoxF function and Notch signaling. In particular, the defects in vSMC coverage of radial arteries in the 6-allele loss retina, presumably reflecting a defect in arterial EC differentiation, resembles the phenotype seen in the *Dll4* vascular knockout [40]. In light of the functional redundancy among *SoxF* family members, it would be of interest to revisit the SoxF/Notch epistasis analysis with mice that carry different combinations of *SoxF* mutations.

### Implications for human disease

At present, the only *SoxF* family member that is unequivocally implicated in human disease is *Sox18*, which is mutated in the hereditary syndrome hypotrichosis-lymphedema-telangiectasia (HLT) [21]. As noted in the Introduction, dominant HLT is caused by truncated derivatives of Sox18 that are presumed to interfere with the function of WT Sox18. Here, we report an edema phenotype in mice with adult onset EC-specific loss of 5/6 or 6/6 *SoxF* alleles, implying that maintenance of the vascular permeability barrier requires ongoing *SoxF* function. These observations suggest that edema in dominant HLT patients and in mice with the analogous *Ra* alleles of *Sox18* could be due, at least in part, to defects in blood vascular permeability, in addition to defects in lymphatic vascular structure and/or function.

Less mechanistically defined, but of greater public health significance, is the positive correlation in multiple genome-wide association studies of non-coding single nucleotide polymorphisms at the *Sox17* locus with intracranial aneurysms [41–44]. This association has received recent experimental support from studies of mice in which EC-specific deletion of *Sox17* combined with pharmacologically-induced chronic hypertension produces cerebrovascular tortuosity, aneurysms, and hemorrhage [45]. This phenotype is accompanied by dilation of cerebral arteries, a thinning of the arterial wall, and a reduction in vSMC coverage. Consistent with that study, the present work provides additional evidence that *Sox17* promotes vSMC coverage and that SoxF activity is required not only for vascular development but also for maintenance. It is tempting to speculate that, in the context of human vascular disease, the compensatory actions of *Sox7* and/or *Sox18* may reduce the deleterious effect of risk-associated *Sox17* alleles.

The most recent connection between human disease and *SoxF* genes comes from an analysis of *Sox17* in tumor ECs [46]. In two mouse models of implantable tumor growth and metastasis, tumor ECs were observed to express *Sox17* at elevated levels; genetic deletion or over-expression of *Sox17* was found to suppress or enhance, respectively, tumor growth and metastasis; and, in a small series, elevated levels of *Sox17* were seen in tumor ECs in the majority of high grade human glioblastomas, but not in low grade glioblastomas or in normal brain tissue. The data suggest that Sox17-mediated transcriptional regulatory patterns that are normally active during vascular development are reactivated in the context of tumor angiogenesis. Thus, a deeper understanding of the regulation of *Sox17* gene expression might provide a basis for the development of novel anti-angiogenic cancer therapies.

## Materials and Methods

### Gene targeting

To create the *Sox17* ^*CKO*^ targeting construct, *loxP* sites were inserted (1) within the first coding exon 5’ of the initiator methionine codon, and (2) 3’ of an SV40 polyadenylation signal that was inserted immediately 3’ of the 3’ UTR. The following DNA segments were added immediately downstream of the 3’ *loxP* site: (1) the coding region for human placental alkaline phosphatase (AP), and (2) a *phosphoglycerate kinase (PGK)-Neo* cassette flanked by *frt* sites (*FNF*). An *EcoR* I site was inserted adjacent to 5’ *loxP* site to facilitate analysis by Southern blotting. In the targeting vector, the 5’ arm extended to an *EcoR* V site ~6 kb upstream of the 5’ *loxP* site, and the 3’ arm extended to a *Hind* III site ~3 kb downstream of the *Sox17* 3’ UTR. (S1A Fig)

PCR primers (F, forward; R, reverse) for distinguishing the WT and *Sox17* ^*CKO*^ alleles are:

Fwt 5′-CAGGTGAAGACACAAACCAGCATC-3′,

Fcko 5′-GAGCTATTCCAGAAGTAGTGAGGAG-3′,

R 5′-AGACTCTGGTTTTCCTGCCTAGTG-3′.

The *Sox17* ^*AP*^ (null) allele, obtained by Cre-mediated recombination, can be genotyped using primers:

F 5′-TTTACGAGTTCCTCTGGGCATCTCCCAGC-3′

R 5′-CCCAGGAAGATGATGAGGTTCTTG-3′.

To create the *Sox7* ^*CKO*^ targeting construct, *loxP* sites were inserted (1) within the first exon 5’ of the initiator methionine codon, and (2) 3’ of an SV40 polyadenylation signal that was inserted immediately 3’ of the 3’ UTR. The following DNA segments were added immediately downstream of the 3’ *loxP* site: (1) the coding region for CFP with a C-terminal 3×HA tag, and (2) a *phosphoglycerate kinase (PGK)-Neo* cassette flanked by *frt* sites (*FNF*), with a third *loxP* site was inserted immediately 5’ of the second *frt* site. *Bgl* II and *EcoR* V sites were inserted, respectively, adjacent to first and third *loxP* sites to facilitate analysis by Southern blotting. In the targeting vector, the 5’ arm extended to an *Sph* I site ~5 kb upstream of the 5’ *loxP* site, and the 3’ arm extended to a *BamH* I site ~5 kb downstream of the *Sox7* 3’ UTR. (S2A Fig)

PCR primers for distinguishing the WT, *Sox7* ^*CKO*^, *Sox7* ^*-*^ alleles are:

F 5′-AAGCCCGAGGCTGCTGGCCAAGTTGGAC-3′,

Rwt+cko 5′-ACACTCCAGTCCCTCGGTCCACGGATAG-3′,

Rnull 5′-GCCACTGCATACTGCTACACCACTCAAG-3′.

Each targeting construct carried a herpes simplex virus *thymidine kinase* gene beyond the 3’ or 5’ homology arm for negative selection in embryonic stem (ES) cells. The targeting constructs were linearized and electroporated into R1 ES cells, and after positive/negative selection with G418 and ganciclovir, ES colonies were screened by Southern blot hybridization. Correctly targeted ES cell clones with a normal karyotype were injected into blastocysts to generate chimeric founders. Germline transmission was confirmed by Southern blot hybridization. Mice carrying the *Sox17* ^*CKO*^ and *Sox7* ^*CKO*^ alleles were crossed to germline *Flp* mice (JAX 003800) to excise the *frt-PGK-Neo-frt* (*FNF*) cassette.

### Mouse lines

The following transgenic mouse alleles were used: *Sox17* ^*CKO*^ (JAX 007686, to generate unmarked *Sox17* ^*-*^ cells), *Sox18* ^*-*^ [22], a kind gift of Dr. Mathias Francois, *Ndp* ^*KO*^ (JAX 012287) [8], *Nrp1* ^*CKO*^ (JAX 005247), *Sox2-Cre* (JAX 014094), *Tie2-Cre* (JAX 008863), *Pdgfb-CreER* [47], *Hprt* ^*LSL-tdT*^ [30]. Mice were handled, housed, and studied according to the guidelines of the Institutional Animal Care and Use Committee (IACUC) of the Johns Hopkins Medical Institutions, which also approved the protocol MO13M469 under which this work was conducted. Embryos were sacrificed at E10.5 by immersion in fixative. Postnatal mice were sacrificed by isolfluorane inhalation, using a saturating level of isofluorane in the atmosphere or by deep ketamine/xylazine anesthesia (100 mg/kg ketamine and 10 mg/kg xylazine) followed by intracardiac perfusion.

### Antibodies

Antibodies used in this study were as follows: goat anti-human Sox7 (AF2766, R and D Systems); rabbit anti-mouse Sox17 (generated against the full length mouse Sox17 by immunizing rabbit with recombinant Sox17 protein) [9]; rabbit anti-mouse Sox18 (generated against the full length mouse Sox18 by immunizing rabbit with recombinant Sox18 protein; this study); rabbit anti-3×HA (generated against T7 gene 10-3HA fusion protein); rabbit anti-human placental alkaline phosphatase (AHP537, AbD Serotec); rat anti-ICAM2 (553326, BD Biosciences), rat anti-Endomucin (sc-65495, Santa Cruz), mouse anti-alpha smooth muscle actin, clone 1A4, Cy3 conjugate (C6198, Sigma-Aldrich), mouse anti-Claudin-5, Alexa 488 conjugate (352588, Invitrogen), rabbit anti-pimonidazole (PAb2627, Hypoxyprobe), goat anti-Dll4 (AF1389, R and D Systems), goat anti-Tie2 (AF762, R&D Systems); rabbit anti-NG2 (AB5320, Millipore); rat anti-Flk-1/ VEGF-R2 (555307, BD Biosciences); goat anti-Flt-4/ VEGF-R3 (AF743, R and D Systems); rat anti-PLVAP/ MECA-32 (553849, BD Biosciences); goat anti-mouse LYVE-1 (AF2125, R&D Systems); Texas Red streptavidin (SA-5006, Vector Laboratories). AlexFluor-labeled secondary antibodies and GS lectin (Isolectin GS-IB4) were from Invitrogen. Primary antibodies were used at 1:100 to 1:500 dilution for commercial antibody, and at 1:2,000 to 1:10,000 dilution for anti-3xHA, anti-Sox17 and anti-Sox18.

### Preparation and administration of 4HT and tamoxifen, tissue processing, histochemistry, and immunostaining

4HT and tamoxifen were dissolved in sunflower seed oil as described [48]. For pups and adults, 50 μl 4HT and 50–100 μl tamoxifen, respectively, were injected by the intraperitoneal (IP) route.

For alkaline phosphatase (AP) histochemistry, whole-mount embryos were fixed in phosphate buffered saline (PBS) containing 4% paraformaldehyde (PFA) overnight, and then processed and stained with nitroblue tetrazolium/5-bromo-4-chloro-indolyl phosphate (NBT/BCIP) as described [49].

For retinal vasculature, intact eyes were fixed in PBS with 4% PFA for 0.5 to 1 hour prior to dissection. For postnatal tissue other than retina, deeply anesthetized mice were perfused via an intra-cardic route with 10 ml PBS with 0.3 mg/ml sulfo-NHS-LC-Biotin (Thermo Scientific 21335), followed by ~20 ml 2% PFA. Dissected organs were post-fixed with 1% PFA for 4–6 hours, wash with PBS for 4–6 hours, then embedded in 3% agarose, and 150 μm sections were cut on a vibratome. For embryonic vasculature, embryos were fixed in 2–4% PFA overnight, and washed with PBS for 4–6 hours.

For immunostaining, whole mount retinas, tissue sections, and embryos were incubated 1–7 days in primary antibodies diluted in PBSTC (PBS + 0.3% Triton + 0.1mM CaCl_2_) + 10% normal goat/donkey serum, washed in PBSTC for 6–8 hours, and incubated 1–3 days in secondary antibodies diluted in PBSTC + 10% normal goat/donkey serum. 0.25% mouse serum was included as a competitive blocker when rat primary antibody was used. Retinas, vibratome sections, and embryos were washed in PBSTC and flat-mounted in Fluoromount G (EM Sciences, 17984–25). All immersion fixation, washing, and immunostaining steps were carried out at 4°C. Images were captured using a Zeiss LSM700 confocal microscope, and processed with ImageJ, Photoshop, and Illustrator software.

### Labeling of patent blood vessels and Hypoxyprobe analysis

For GS-lectin perfusion in the living mouse, 50 μl 0.33 mg/ml Alexa-Fluor 595-conjugated GS-lectin in PBS with 1 mM CaCl_2_ were delivered via intra-cardiac injection 15–30 minutes before harvest [50]. For hypoxia analysis, 2.5 mg Hypoxyprobe in 50 μl PBS was injected IP 1 hour before harvest [28]. Following intracardiac perfusion with phosphate buffered saline (PBS) containing 4% paraformaldehyde (PFA) retinas were processed for immunostaining with anti-pimonidazole antibodies as described above.

### Miles Assay

To test vascular permeability of adult mice with 6-allele loss, the Miles assay was performed as described [51]. In brief, 100 μl 0.6% Evans Blue was injected via the tail vein. Thirty minutes later, lung, liver and muscle were harvested, and Evans Blue was extracted in 500 μl formamide at 55°C for 48–72 h. The absorbance was measured at 600 nm.

### Quantification of pixel intensities

Images were exported to ImageJ and converted to an 8-bit format. A median filter (radius = 0.5 pixels) was applied, and the background (determined with a rolling circle with a 50 pixel radius) was subtracted from all channels. After splitting the Sox7 and Sox17 channels, the “Co-localization Finder” within the “Analyze” menu (installed as a plug-in) was used to produce a scatter-plot of pixel intensities by choosing the Sox17 image as red (x-axis) and the Sox7 image as green (y-axis). Additional details are available at: http://imagej.net/Image_Intensity_Processing.

## Supporting Information

S1 Fig Structure of the Sox17^CKO^ allele and expression of the AP reporter. (A) Targeted recombination and the resulting structure of the *Sox17* ^*CKO*^ allele. Cre-mediated recombination deletes the *Sox17* coding sequences and activates AP. E, *EcoR* I. (B) *Sox17* is expressed in ECs as determined by whole mount AP histochemistry of a *Sox17* ^*AP/+*^ E10.5 embryo (right).
(PDF)

S2 Fig Structure of the Sox7^CKO^ allele and expression of the CFP-3HA reporter. (A) Targeted recombination and the resulting structure of the *Sox7* ^*CKO*^ allele. Cre-mediated recombination prior to *PGK-Neo* excision deletes the *Sox7* coding sequences and 3×HA tagged CFP (*Sox7* ^*-*^; bottom). Cre-mediated recombination after *PGK-Neo* excision deletes *Sox7* coding sequences and activates 3×HA tagged CFP (*Sox7* ^*CFP-3xHA*^; second from bottom). EV, *EcoR* V; B, *Bgl* II. (B) *Sox7* is expressed in ECs as determined by anti-HA immunostaining in flat-mounted retinas from P10 *Sox7* ^*CFP-3xHA/+*^ mice. HA signal is not detected in mice carrying the *Sox7* ^*-*^, or *Sox7* ^*CKO*^ alleles. Scale bar, 200 μm. (C) *Sox7* expression varies in different tissues and organs as determined by anti-HA and anti-Sox7 staining in WT (upper panels) and *Sox7* ^*CFP-3xHA/+*^ (bottom panels) mice at P2. Scale bar, 200 μm. (D) Flat mount retinas show that *Sox17* and *Sox7* are expressed in the adult retina, as determined by anti-AP and anti-HA staining of *Sox17* ^*AP*^ and *Sox7* ^*CFP-3xHA*^ retinas, respectively. Scale bar, 200 μm.
(PDF)

S3 Fig Sox7 is required in Tie2-expressing cells during embryonic development. (A) Embryonic lethality in *Sox7* ^*-/-*^, *Sox7* ^*CKO/CKO*^ *;Tie2-Cre* and *Sox7* ^*+/-*^ *;Sox17* ^*+/-*^ embryos. (B) Left, whole mount E10.5 control and *Sox7* ^*CKO/CKO*^ *;Tie2-Cre* embryos stained with anti-Icam2, anti-Sox7 and anti-Sox17. *Sox7* is ubiquitously expressed in the vasculature, but *Sox17* expression is absent from cardiac vessels. Higher magnification images of the heart (red square) is shown in the right panels; Sox17+ ECs in vasculature adjacent to the heart are indicated by arrows. Scale bar, 200 μm. (C) Left, dorsal aortas from control and *Sox7* ^*CKO/CKO*^ *;Tie2-Cre* E10.5 embryos are outlined by dashed lines, and the width of the aorta is highlighted by the double-headed arrows. Right, the ratio of the left dorsal aorta to the right dorsal aorta (Y-axis) plotted against the width of left dorsal aorta (X-axis). Red circles represent WT, blue circles represent *Sox7* ^*CKO/+*^ *;Tie2-Cre*, and green circles represent *Sox7* ^*CKO/CKO*^ *;Tie2-Cre*. Black arrows indicate two *Sox7* ^*CKO/CKO*^ *;Tie2-Cre* embryos with more severe growth retardation. The minimally growth retarded *Sox7* ^*CKO/CKO*^ *;Tie2-Cre* embryos show a relatively smaller left dorsal aorta with normal coverage by vSMCs. A student’s t-test comparison of the ratio of the left vs. right dorsal aortas gives a P-value of 9x10^-3^ for the comparison of WT vs. *Sox7* ^*CKO/CKO*^ *;Tie2-Cre*, and a P-value of 1.7x10^-6^ for the same comparison if the two growth retarded embryos (arrows) are omitted. Scale bar, 200 μm.
(PDF)

S4 Fig Loss of both Sox17 and Sox18 causes severe defects in retinal and cerebellar angiogenesis. (A, B) Retina flat mounts show a hyperplasia phenotype in *Sox17* ^*CKO/CKO*^ *;Sox18* ^*-/-*^ *;Pdgfb-CreER* vasculature at P6 (A, 4-allele loss), but not in *Sox17* ^*CKO/+*^ *;Sox18* ^*-/-*^ *;Pdgfb-CreER* or *Sox17* ^*CKO/-*^ *;Sox18* ^*+/-*^ *;Pdgfb-CreER* vasculature at P8 (B, 3-allele loss). Mice were treated with ~20 μg 4HT at P1. Scale bar, 200 μm. (C) Retina flat mounts from *Sox17* ^*CKO/+*^ *;Sox18* ^*-/-*^ *;Pdgfb-CreER*, *Sox17* ^*CKO/-*^ *;Sox18* ^*+/-*^ *;Pdgfb-CreER* and *Sox17* ^*CKO/-*^ *;Sox18* ^*-/-*^ *;Pdgfb-CreER* without (upper panel) and after (bottom panel) tamoxifen treatment (producing 3- and 4-allele loss). No vascular defects were seen in the *Sox17* ^*CKO/-*^ *;Sox18* ^*-/-*^ *;Pdgfb-CreER* retina when 2–2.5 mg of tamoxifen was given at P21 and P28. Anti-AP staining shows nearly complete recombination of *Sox17* ^*CKO*^ mediated by *Pdgfb-CreER*. Scale bar, 200 μm. (D) Retina flat mounts show vein enlargment (arrows) and hyperplasia in *Sox17* ^*CKO/CKO*^ *;Sox18* ^*-/-*^ *;Pdgfb-CreER* vasculature at P12. Mice were treated with ~50 μg 4HT at P4. Scale bar, 200 μm. (E) Cerebellum sections from *Sox18* ^*+/-*^ control and *Sox17* ^*CKO/-*^ *;Sox18* ^*-/-*^ *;Pdgfb-CreER* at P5, following 20 μg 4HT at P1. Vascular sprouting (arrows) was seen in the *Sox17* ^*CKO/-*^ *;Sox18* ^*-/-*^ *;Pdgfb-CreER* cerebellum. Scale bar, 200 μm.
(PDF)

S5 Fig Sox7, Sox17 and Sox18 EC-specific triple KO (6-allele loss) in adulthood does not alter vascular permeability when measured acutely. (A) Retina flat mounts from P33 control *Sox18* ^*+/-*^ (left panels) and *Sox7* ^*CKO/-*^ *;Sox17* ^*CKO/CKO*^ *;Sox18* ^*-/-*^ *;Pdgfb-CreER* (6-allele loss; middle and right panels) mice treated with 2–3 mg tamoxifen at P21, P25, and P29. Endomucin labels veins and capillaries, smooth muscle actin labels arteries, and anti-HA and anti-Sox17 staining indicates CreER-mediated recombination efficiency. Vascular anatomy is normal in the 6-allele loss retina. Scale bar, 200 μm for left and middle panels, 500 μm for right panels. (B) Intracardiac perfusion with sulfo-NHS-biotin shows that the blood-brain barrier is intact in *Sox7* ^*CKO/-*^ *;Sox17* ^*CKO/CKO*^ *;Sox18* ^*-/-*^ *;Pdgfb-CreER* cerebral cortex at P40, following 2–3 mg tamoxifen at P21, P25 and P29. Biotin labeling of kidney parenchyma serves as a positive control for sulfo-NHS-biotin perfusion. Plasmalemma vesicle–associated protein (PLVAP, magenta), a component of endothelial fenestrations, is normally absent from brain ECs; Claudin5 (green), a tight junction protein, is expressed by brain ECs. Scale bar, 200 μm. (C) Vascular permeability of *Sox7* ^*CKO/-*^ *;Sox17* ^*CKO/CKO*^ *;Sox18* ^*-/-*^ *;Pdgfb-CreER* was assessed by Evans Blue extravasation (Miles assay) in different tissues. 50–100 mg tissue was collected and incubated with 500 μl formamide, ~30 min after intravenous injection of 100 μl 0.6% Evans Blue. Optical density was measured at 600 nm. A = 6-allele loss. B = *Sox18* ^*+/-*^; C = 1 copy of *Sox7*. See Fig 4A for a definition of genotypes.
(PDF)

S6 Fig Specificity of the Pdgfb-CreER transgene in skin vasculature. A male *Hprt* ^*LSL-tdT/Y*^ *;Pdgfb-CreER* mouse was treated with 2–3 mg IP tamoxifen at P21, P23, and P25. At P29, flat mounts of skin from the ear and abdominal wall was analyzed for expression of the nuclear-localized tdTomato reporter following Cre-mediated excision of the *loxP-stop-loxP* segment. tdTomato is not present in lymphatic vessels, visualized with Lyve1, but is present in virtually all blood vascular endothelial cells, visualized with CD31. In the abdominal wall image there is background red fluorescence from hair follicles. Scale bar, 200 um
(PDF)
