# Supplementary figures and images for: Sox7, Sox17, and Sox18 Cooperatively Regulate Vascular Development in the Mouse Retina
Source: PLoS One. 2015 Dec 2;10(12):e0143650. doi: 10.1371/journal.pone.0143650 (PMC4667919; doi:10.1371/journal.pone.0143650)

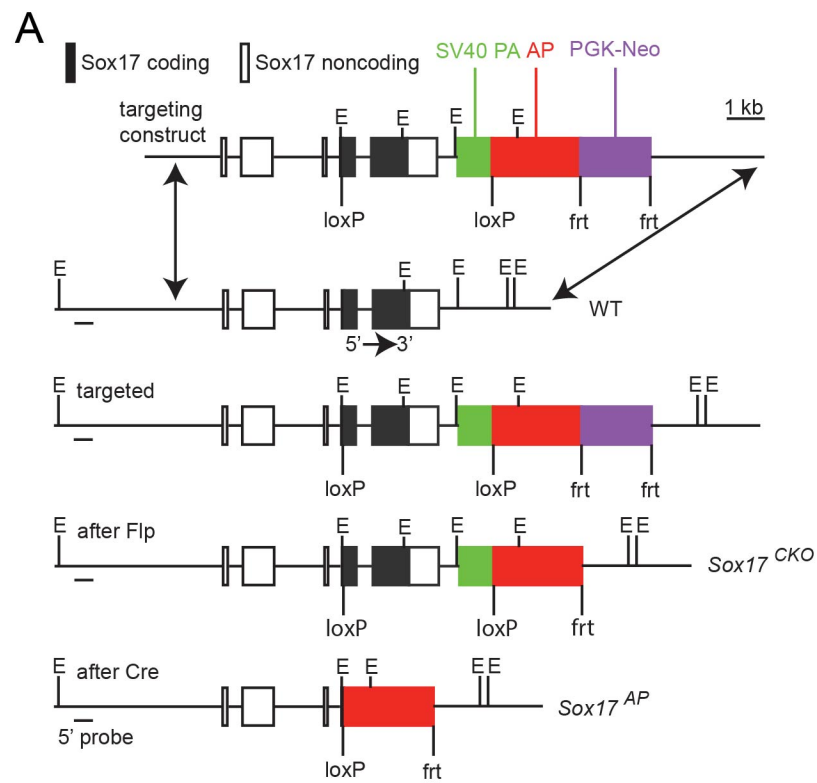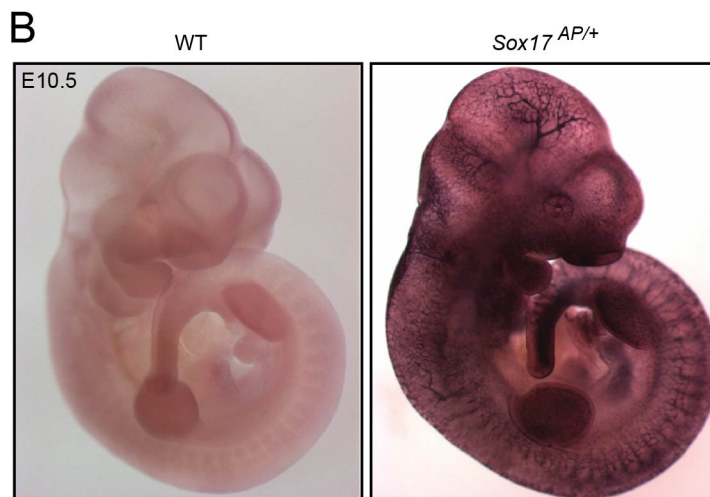

Zhou et al, Figure S1

Supplement: S1 Fig — (A) Targeted recombination and the resulting structure of the Sox17 CKO allele. Cre-mediated recombination deletes the Sox17 coding sequences and activates AP. E, EcoR I. (B) Sox17 is expressed in ECs as determined by whole mount AP histochemistry of a Sox17 AP/+ E10.5 embryo (right). (PDF) [file pone.0143650.s001.pdf]

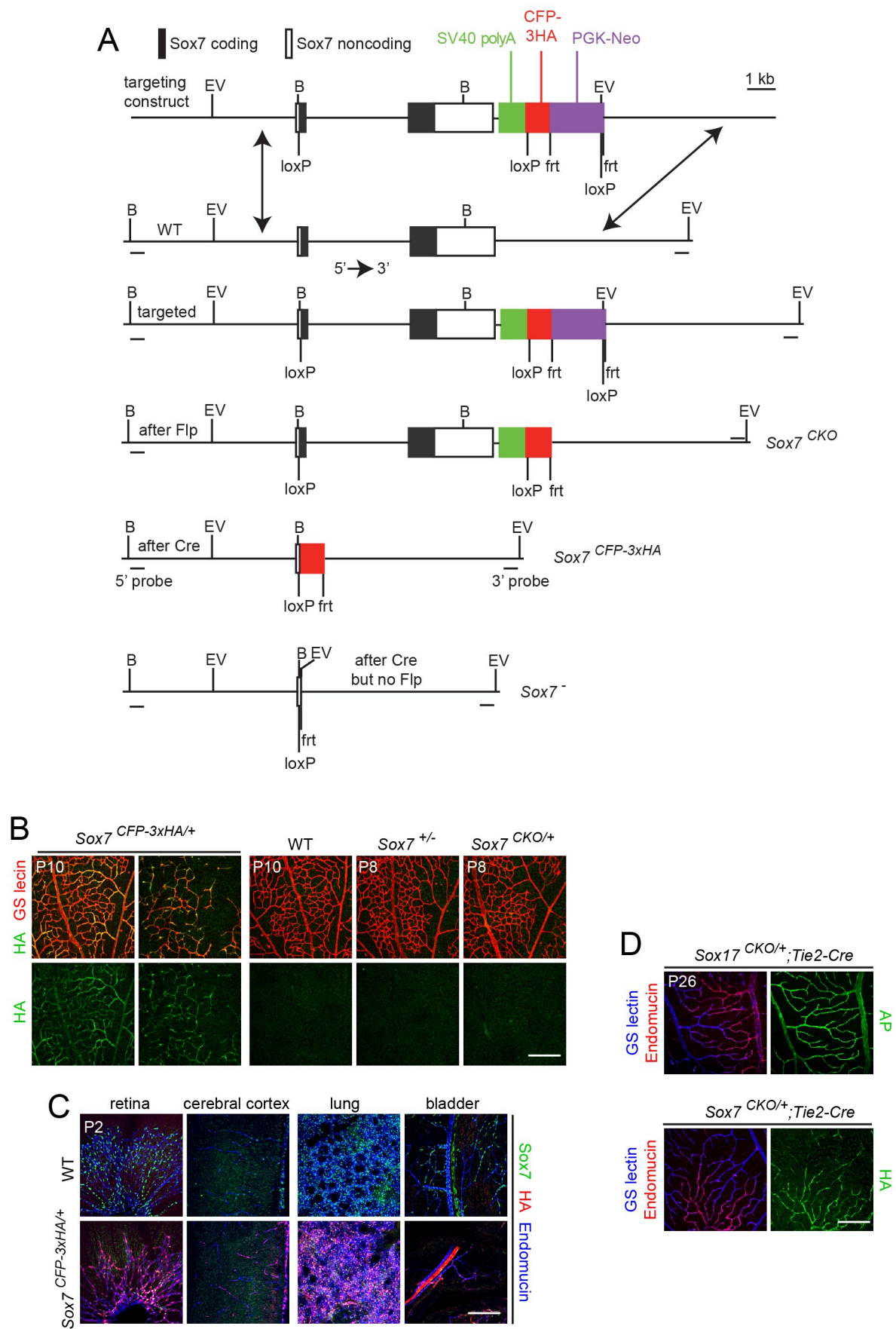

Zhou et al, Figure S2

Supplement: S2 Fig — (A) Targeted recombination and the resulting structure of the Sox7 CKO allele. Cre-mediated recombination prior to PGK-Neo excision deletes the Sox7 coding sequences and 3×HA tagged CFP (Sox7 -; bottom). Cre-mediated recombination after PGK-Neo excision deletes Sox7 coding sequences and activates 3×HA tagged CFP (Sox7 CFP-3xHA; second from bottom). EV, EcoR V; B, Bgl II. (B) Sox7 is expressed in ECs as determined by anti-HA immunostaining in flat-mounted retinas from P10 Sox7 CFP-3xHA/+ mice. HA signal is not detected in mice carrying the Sox7 -, or Sox7 CKO alleles. Scale bar, 200 μm. (C) Sox7 expression varies in different tissues and organs as determined by anti-HA and anti-Sox7 staining in WT (upper panels) and Sox7 CFP-3xHA/+ (bottom panels) mice at P2. Scale bar, 200 μm. (D) Flat mount retinas show that Sox17 and Sox7 are expressed in the adult retina, as determined by anti-AP and anti-HA staining of Sox17 AP and Sox7 CFP-3xHA retinas, respectively. Scale bar, 200 μm. (PDF) [file pone.0143650.s002.pdf]

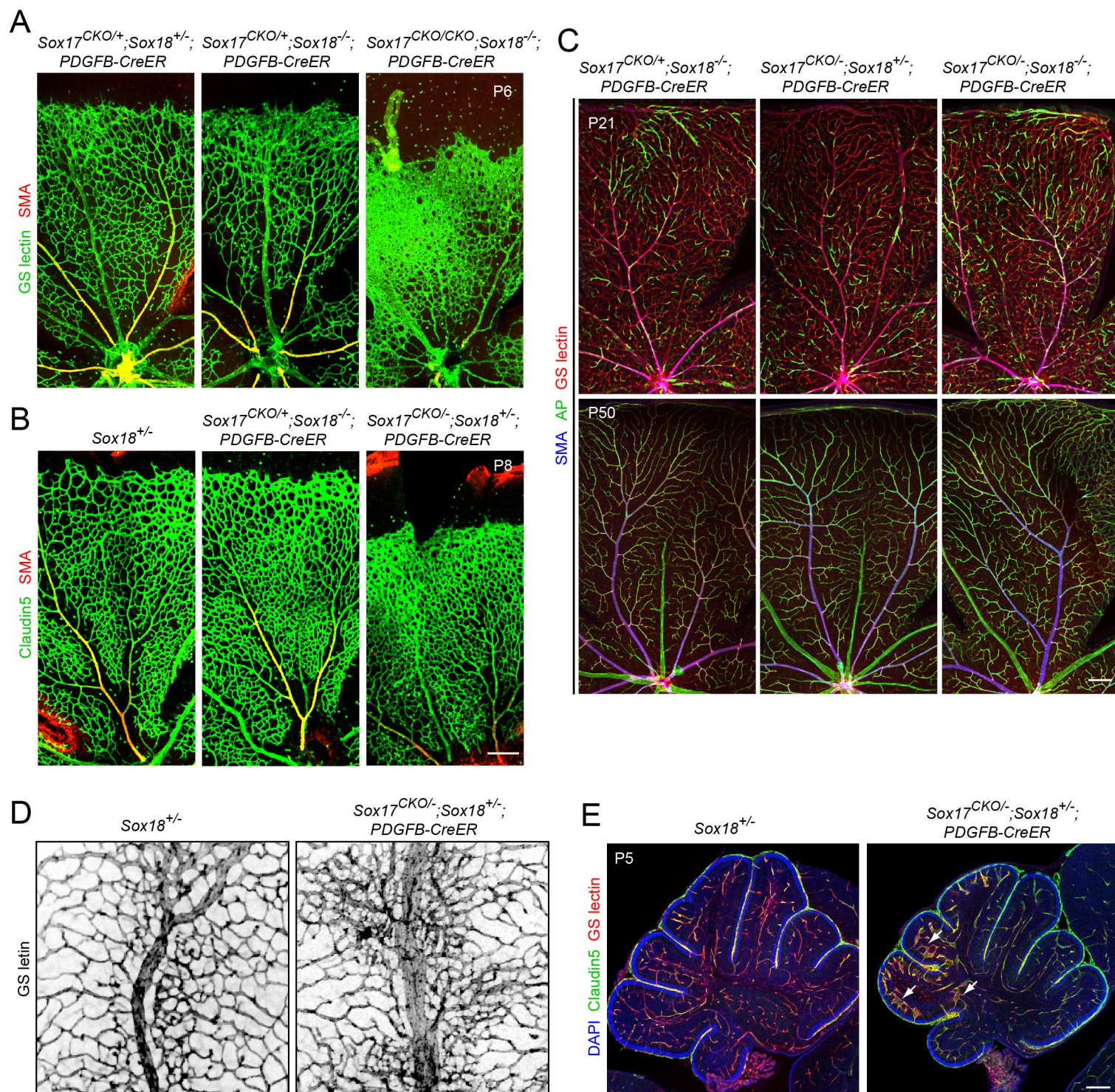

Zhou et al, Figure S4

Supplement: S4 Fig — (A, B) Retina flat mounts show a hyperplasia phenotype in Sox17 CKO/CKO ;Sox18 -/- ;Pdgfb-CreER vasculature at P6 (A, 4-allele loss), but not in Sox17 CKO/+ ;Sox18 -/- ;Pdgfb-CreER or Sox17 CKO/- ;Sox18 +/- ;Pdgfb-CreER vasculature at P8 (B, 3-allele loss). Mice were treated with ~20 μg 4HT at P1. Scale bar, 200 μm. (C) Retina flat mounts from Sox17 CKO/+ ;Sox18 -/- ;Pdgfb-CreER, Sox17 CKO/- ;Sox18 +/- ;Pdgfb-CreER and Sox17 CKO/- ;Sox18 -/- ;Pdgfb-CreER without (upper panel) and after (bottom panel) tamoxifen treatment (producing 3- and 4-allele loss). No vascular defects were seen in the Sox17 CKO/- ;Sox18 -/- ;Pdgfb-CreER retina when 2–2.5 mg of tamoxifen was given at P21 and P28. Anti-AP staining shows nearly complete recombination of Sox17 CKO mediated by Pdgfb-CreER. Scale bar, 200 μm. (D) Retina flat mounts show vein enlargment (arrows) and hyperplasia in Sox17 CKO/CKO ;Sox18 -/- ;Pdgfb-CreER vasculature at P12. Mice were treated with ~50 μg 4HT at P4. Scale bar, 200 μm. (E) Cerebellum sections from Sox18 +/- control and Sox17 CKO/- ;Sox18 -/- ;Pdgfb-CreER at P5, following 20 μg 4HT at P1. Vascular sprouting (arrows) was seen in the Sox17 CKO/- ;Sox18 -/- ;Pdgfb-CreER cerebellum. Scale bar, 200 μm. (PDF) [file pone.0143650.s004.pdf]

*Hprt<sup>LSL-tdTr</sup>; PDGFB-CreER*  
(tamoxifen at P21, P23, P25)

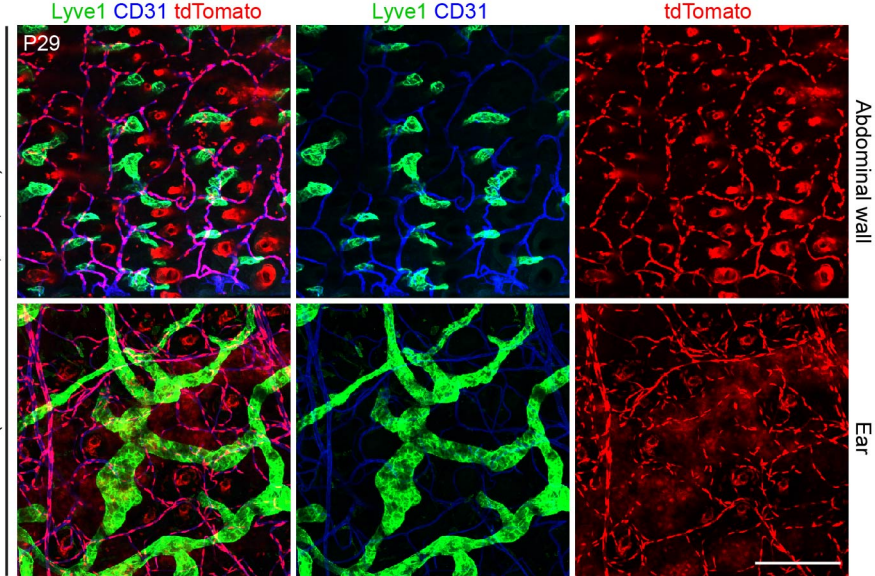

Supplement: S6 Fig — A male Hprt LSL-tdT/Y ;Pdgfb-CreER mouse was treated with 2–3 mg IP tamoxifen at P21, P23, and P25. At P29, flat mounts of skin from the ear and abdominal wall was analyzed for expression of the nuclear-localized tdTomato reporter following Cre-mediated excision of the loxP-stop-loxP segment. tdTomato is not present in lymphatic vessels, visualized with Lyve1, but is present in virtually all blood vascular endothelial cells, visualized with CD31. In the abdominal wall image there is background red fluorescence from hair follicles. Scale bar, 200 um (PDF) [file pone.0143650.s006.pdf]
