# Supplementary material for: Sox7, Sox17, and Sox18 Cooperatively Regulate Vascular Development in the Mouse Retina
Source: PLoS One. 2015 Dec 2;10(12):e0143650. doi: 10.1371/journal.pone.0143650 (PMC4667919; doi:10.1371/journal.pone.0143650)
Supplement: S3 Fig — (A) Embryonic lethality in Sox7 -/-, Sox7 CKO/CKO ;Tie2-Cre and Sox7 +/- ;Sox17 +/- embryos. (B) Left, whole mount E10.5 control and Sox7 CKO/CKO ;Tie2-Cre embryos stained with anti-Icam2, anti-Sox7 and anti-Sox17. Sox7 is ubiquitously expressed in the vasculature, but Sox17 expression is absent from cardiac vessels. Higher magnification images of the heart (red square) is shown in the right panels; Sox17+ ECs in vasculature adjacent to the heart are indicated by arrows. Scale bar, 200 μm. (C) Left, dorsal aortas from control and Sox7 CKO/CKO ;Tie2-Cre E10.5 embryos are outlined by dashed lines, and the width of the aorta is highlighted by the double-headed arrows. Right, the ratio of the left dorsal aorta to the right dorsal aorta (Y-axis) plotted against the width of left dorsal aorta (X-axis). Red circles represent WT, blue circles represent Sox7 CKO/+ ;Tie2-Cre, and green circles represent Sox7 CKO/CKO ;Tie2-Cre. Black arrows indicate two Sox7 CKO/CKO ;Tie2-Cre embryos with more severe growth retardation. The minimally growth retarded Sox7 CKO/CKO ;Tie2-Cre embryos show a relatively smaller left dorsal aorta with normal coverage by vSMCs. A student’s t-test comparison of the ratio of the left vs. right dorsal aortas gives a P-value of 9x10^-3^ for the comparison of WT vs. Sox7 CKO/CKO ;Tie2-Cre, and a P-value of 1.7x10^-6^ for the same comparison if the two growth retarded embryos (arrows) are omitted. Scale bar, 200 μm. (PDF) [file pone.0143650.s003.pdf]

A

Genotype of Parents	Genotype of Progeny	Live	Total	Live/Total Expected ratio	P-Value
Sox7 $^{+/-}$ × *Sox7* $^{+/-}$	*Sox7* $^{-/-}$	0	33	1/4	7.5×10^{-5}
Sox7 $^{CKO/+}$; *Tie2-Cre* × *Sox7* $^{CKO/CKO}$	*Sox7* $^{CKO/CKO}$; *Tie2-Cre*	0	66	1/4	5.7×10^{-9}
Sox7 $^{+/-}$ × *Sox17* $^{+/-}$	*Sox7* $^{+/-}$; *Sox17* $^{+/-}$	1	51	1/4	7.2×10^{-6}

B

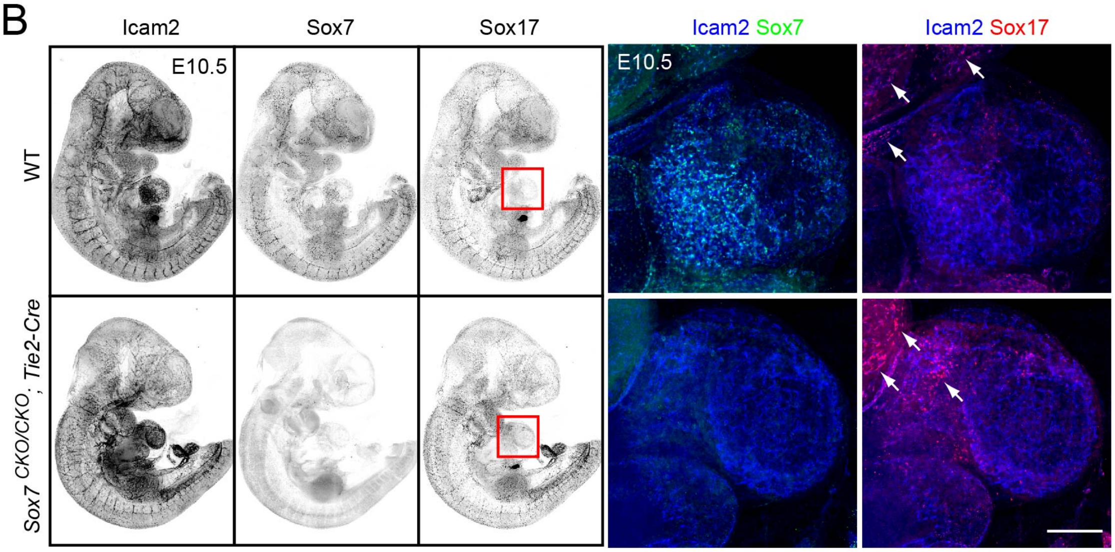

C

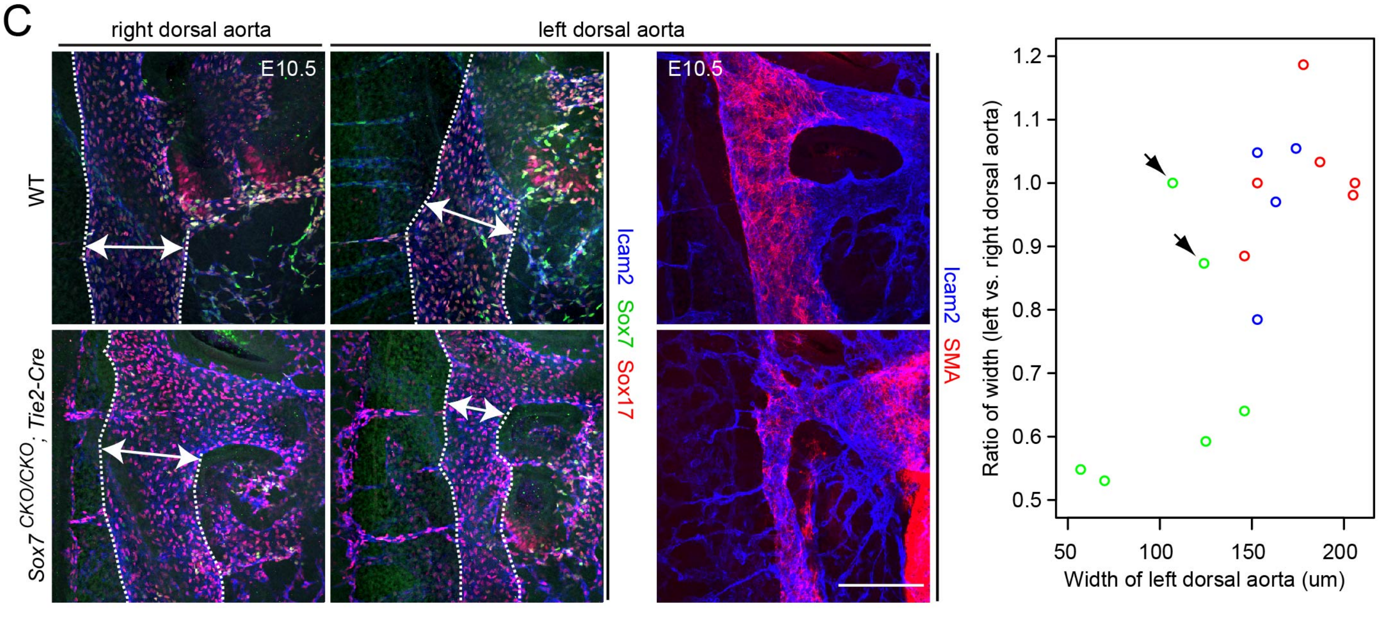

Zhou et al, Figure S3
