# Supplementary material for: Sox7, Sox17, and Sox18 Cooperatively Regulate Vascular Development in the Mouse Retina
Source: PLoS One. 2015 Dec 2;10(12):e0143650. doi: 10.1371/journal.pone.0143650 (PMC4667919; doi:10.1371/journal.pone.0143650)
Supplement: S5 Fig — (A) Retina flat mounts from P33 control Sox18 +/- (left panels) and Sox7 CKO/- ;Sox17 CKO/CKO ;Sox18 -/- ;Pdgfb-CreER (6-allele loss; middle and right panels) mice treated with 2–3 mg tamoxifen at P21, P25, and P29. Endomucin labels veins and capillaries, smooth muscle actin labels arteries, and anti-HA and anti-Sox17 staining indicates CreER-mediated recombination efficiency. Vascular anatomy is normal in the 6-allele loss retina. Scale bar, 200 μm for left and middle panels, 500 μm for right panels. (B) Intracardiac perfusion with sulfo-NHS-biotin shows that the blood-brain barrier is intact in Sox7 CKO/- ;Sox17 CKO/CKO ;Sox18 -/- ;Pdgfb-CreER cerebral cortex at P40, following 2–3 mg tamoxifen at P21, P25 and P29. Biotin labeling of kidney parenchyma serves as a positive control for sulfo-NHS-biotin perfusion. Plasmalemma vesicle–associated protein (PLVAP, magenta), a component of endothelial fenestrations, is normally absent from brain ECs; Claudin5 (green), a tight junction protein, is expressed by brain ECs. Scale bar, 200 μm. (C) Vascular permeability of Sox7 CKO/- ;Sox17 CKO/CKO ;Sox18 -/- ;Pdgfb-CreER was assessed by Evans Blue extravasation (Miles assay) in different tissues. 50–100 mg tissue was collected and incubated with 500 μl formamide, ~30 min after intravenous injection of 100 μl 0.6% Evans Blue. Optical density was measured at 600 nm. A = 6-allele loss. B = Sox18 +/-; C = 1 copy of Sox7. See Fig 4A for a definition of genotypes. (PDF) [file pone.0143650.s005.pdf]

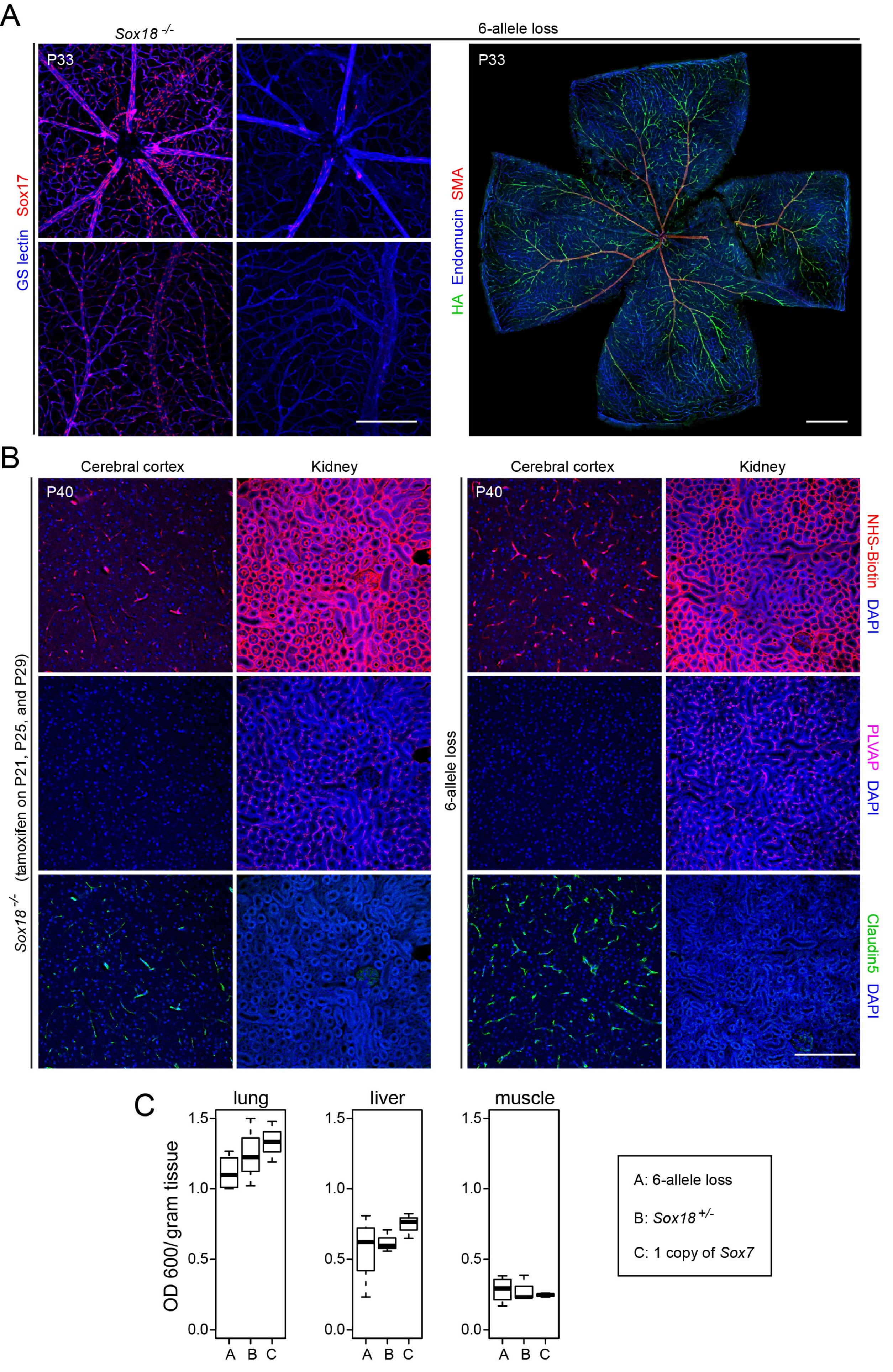

Zhou et al Figure S5
